# Supplementary material for: Quantitative Assessment of Eye Phenotypes for Functional Genetic Studies Using Drosophila melanogaster
Source: G3 (Bethesda). 2016 Mar 18;6(5):1427–37. doi: 10.1534/g3.116.027060 (PMC4856093; doi:10.1534/g3.116.027060)
Supplement: Supplemental Material [file supp_6_5_1427__index.html]

Quantitative Assessment of Eye Phenotypes for Functional Genetic Studies Using Drosophila melanogaster — Supplemental Material 

# Quantitative Assessment of Eye Phenotypes for Functional Genetic Studies Using *Drosophila melanogaster*

## Supplemental Material for Iyer *et al.*, 2016

**Files in this Data Supplement:**

- Supplemental Material - This file contains all Supplemental Figures, Tables, and References. (.pdf, 2,622 KB)
- Table S1 - Stock list of candidate genotypes prioritized from the deficiency screen of modifiers of UAS-*so*. (.pdf, 14 KB)
- Table S2 - Primers used for quantitative real time PCR. (.pdf, 14 KB)
- Figure S1 - Optimization of eye area identification. (.pdf, 201 KB)
- Table S3 - A list of neurodevelopmental genes assessed for eye phenotypes. (.pdf, 37 KB)
- Table S4 - *Drosophila* orthologs of human neurodevelopmental genes and the qualitative rank order of their eye phenotypes. (.pdf, 19 KB)
- Table S5 - Student *t* test comparing eye phenotypes of neurodevelopmental genes with controls. (.pdf, 20 KB)
- Table S6 - Student *t* test comparing phenotypic scores of eye phenotypes at 28°C to that at 30°C. (.pdf, 18 KB)
- Table S7 - Student *t* test comparing the phenotypic scores of modifiers of UAS-*so* with phenotypic scores from UAS-*so* alone. (.pdf, 25 KB)
- Table S8 - Features and limitations of Flynotyper. (.pdf, 14 KB)
- Supplemental References - File contains Supplemental References. (.pdf, 28 KB)
- Figure S9 - Performance of Flynotyper for SEM and bright field microscope images. (.pdf, 195 KB)
- Figure S2 - Eye area localization of images obtained from SEM. (.pdf, 83 KB)
- Figure S3 - User interface for Flynotyper using the ImageJ plugin. (.pdf, 364 KB)
- Figure S4 - Calculation of phenotypic score. (.pdf, 364 KB)
- Figure S5 - Analysis of different categories of eye phenotypes. (.pdf, 20 KB)
- Figure S6 - Phenotypic analysis of fly lines with RNAi mediated knockdown of para (SCN1A in humans). (.pdf, 127 KB)
- Figure S7 - Performance of Flynotyper at different ommatidial counts (N). (.pdf,
- Figure S8 - Performance of Flynotyper at image resolutions is shown. (.pdf, 63 KB)
- Figure S10 - A test for sensitivity of Flynotyper and its ability to distinguish between different classes of phenotypes. (.pdf, 180 KB)
- Figure S11 - Validation of Flynotyper for images obtained from independent studies (Example 1). (.pdf, 148 KB)
- Figure S12 - Validation of Flynotyper for images obtained from independent studies (Example 2). (.pdf, 212 KB)
- Figure S13 - Suppression of UAS-i(CTG)480 toxicity by transgenic expression of ABP1 peptide. (.pdf, 81 KB)
- Figure S14 - A genetic screen for interactors of Egfr using P-element insertions. (.pdf, 152 KB)
- Figure S15 - Flynotyper analysis of SEM images to identify modifiers of *sine oculis*. (.pdf, 145 KB)
- Figure S16 - Flowchart depicting the utility of Flynotyper. (.pdf, 12 KB)
