# Supplementary material for: Quantitative Assessment of Eye Phenotypes for Functional Genetic Studies Using Drosophila melanogaster
Source: G3 (Bethesda). 2016 Mar 18;6(5):1427–37. doi: 10.1534/g3.116.027060 (PMC4856093; doi:10.1534/g3.116.027060)
Supplement: Supplemental Material [file supp_g3.116.027060_027060SM.pdf]

SUPPLEMENTARY INFORMATION

Quantitative assessment of eye phenotypes for functional genetic studies using *Drosophila melanogaster*

Janani Iyer[*, †], Qingyu Wang[‡, †], Thanh Le[‡, †], Lucilla Pizzo[*], Sebastian Grönke[§], Surendra S. Ambegaokar[**], Yuzuru Imai[††], Ashutosh Srivastava[*], Beatriz Llamusí Troisí[‡‡, §§], Graeme Mardon[***], Ruben Artero[‡‡], George R. Jackson[†††], Adrian M. Isaacs[‡‡‡], Linda Partridge[§], Bingwei Lu[§§§], Justin P. Kumar[****], Santhosh Girirajan[*, ‡, ††††]

Table of Contents

Supplementary Figures 3

Figure S1. Optimization of eye area identification. 3

Figure S2. Eye area localization of images obtained from SEM. 4

Figure S3. User interface for Flynotyper using the ImageJ plugin. 5

Figure S4. Calculation of phenotypic score. 6

Figure S5. Analysis of different categories of eye phenotypes. 7

Figure S6. Phenotypic analysis of fly lines with RNAi mediated knockdown of para (*SCN1A* in humans). 8

Figure S7. Performance of Flynotyper at different ommatidial counts (N). 9

Figure S8. Performance of Flynotyper at image resolutions is shown. 10

Figure S9. Performance of Flynotyper for SEM and bright field microscope images. 11

Figure S10. A test for sensitivity of Flynotyper and its ability to distinguish between different classes of phenotypes. 12

Figure S11. Validation of Flynotyper for images obtained from independent studies (Example 1). 13

Figure S12. Validation of Flynotyper for images obtained from independent studies (Example 2). 14

Figure S13. Suppression of UAS-i(CTG)480 toxicity by transgenic expression of ABP1 peptide. 16

Figure S14. A genetic screen for interactors of Egfr using P-element insertions. 17

Figure S15. Flynotyper analysis of SEM images to identify modifiers of *sine oculis*. 18

Figure S16. Flowchart depicting the utility of Flynotyper. 19

Supplementary Tables 20

Table S1. Stock list of candidate genotypes prioritized from the deficiency screen of modifiers of UAS-*so* 20

Table S2. Primers used for quantitative real time PCR 21

Table S3. A list of neurodevelopmental genes assessed for eye phenotypes 22

Table S4. *Drosophila* orthologs of human neurodevelopmental genes and the qualitative rank order of their eye phenotypes 26

Table S5. Student *t* test comparing eye phenotypes of neurodevelopmental genes with controls 27

Table S6. Student *t* test comparing phenotypic scores of eye phenotypes at 28°C to that at 30°C 28

Table S7. Student *t* test comparing the phenotypic scores of modifiers of UAS-*so* with phenotypic scores from UAS-*so* alone 29

Table S8. Features and limitations of Flynotyper 31

References 32

Supplementary Figures

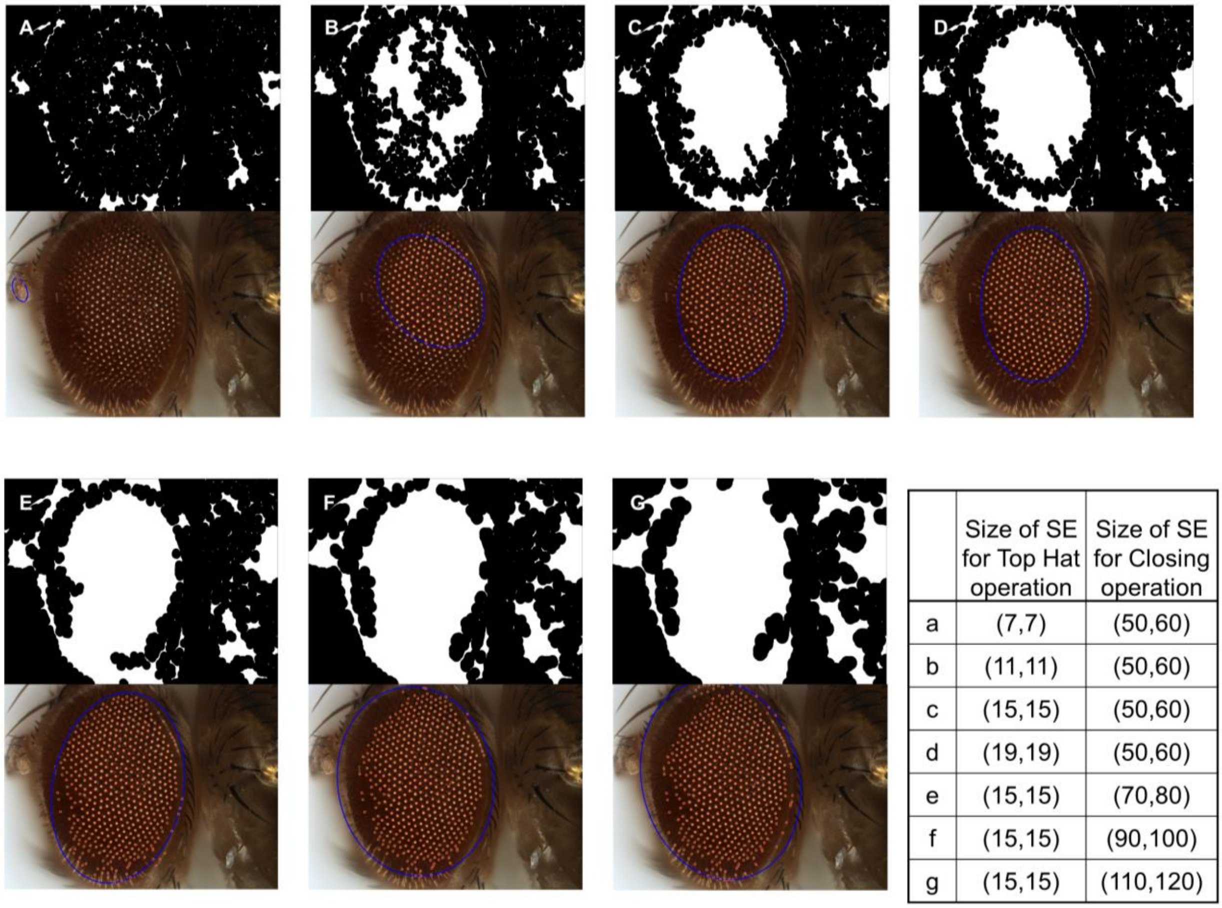

	Size of SE for Top Hat operation	Size of SE for Closing operation
a	(7,7)	(50,60)
b	(11,11)	(50,60)
c	(15,15)	(50,60)
d	(19,19)	(50,60)
e	(15,15)	(70,80)
f	(15,15)	(90,100)
g	(15,15)	(110,120)

Figure S1. Optimization of eye area identification.
Different combinations of structuring elements (SE) sizes were tested for the Top Hat and Closing transformation operations to optimize the accuracy of eye area localization (**A-G**). All sizes of structuring elements used for the transformation operations are shown in the table.

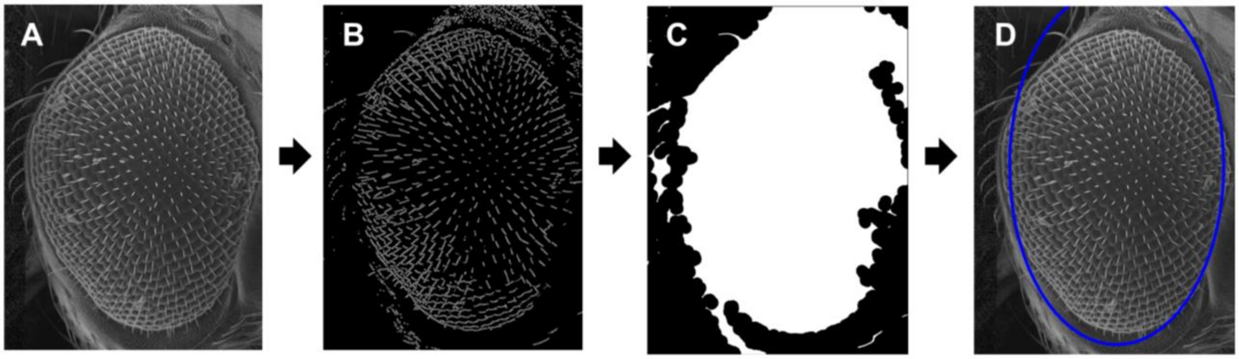

Figure S2. Eye area localization of images obtained from SEM.
Eye localization in SEM image is performed by **(A)** first applying a thresholding operation to the grayscale SEM image, which enables **(B)** better separation of the eye from the background followed by **(C)** edge detection to detect ommatidial cluster region and **(D)** finally closing operation is performed to localize the eye area.

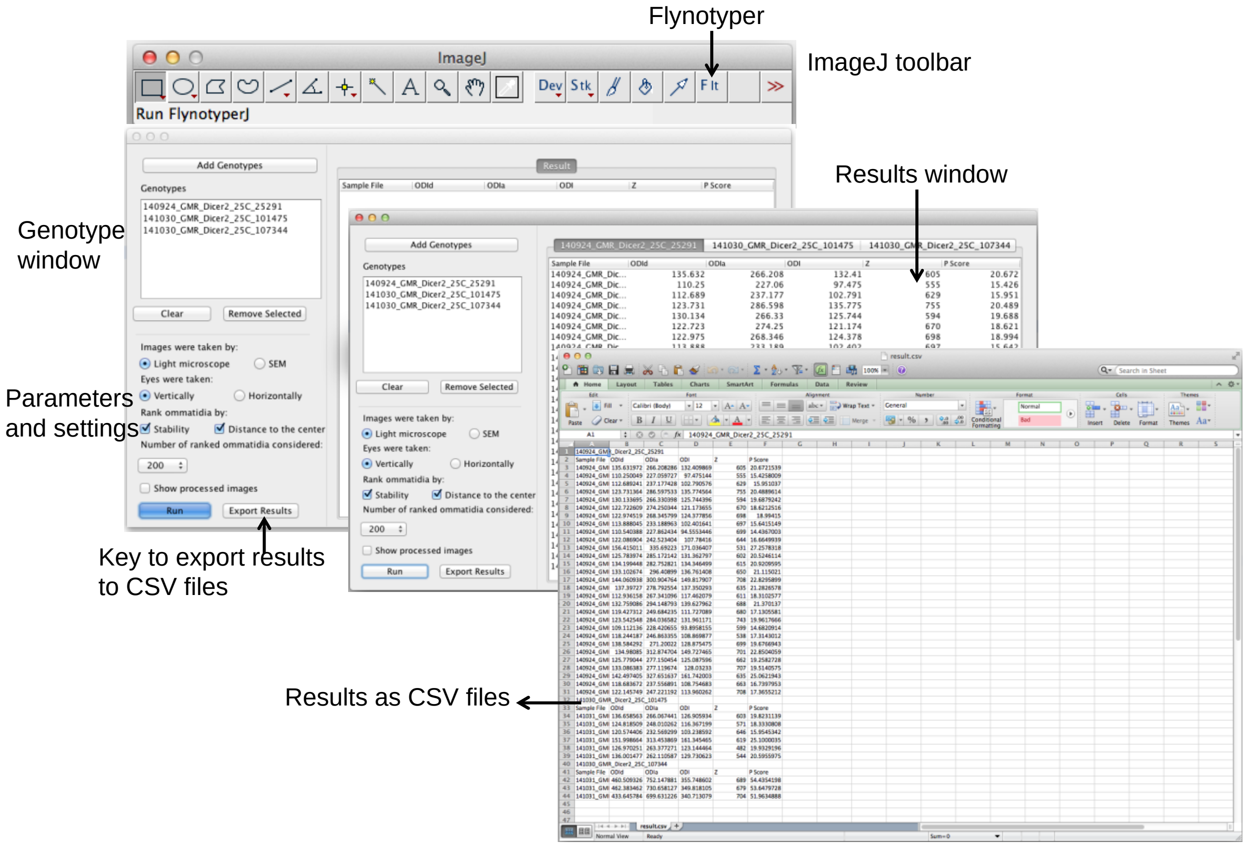

Figure S3. User interface for Flynotyper using the ImageJ plugin.
User interface for imageJ plugin for using Flynotyper shows different components of the software that are user friendly. The parameters and setting can be changed based on the type of microscope used (SEM or bright field), number of ranked ommatidia to be considered for phenotypic score calculation, type of image (vertical or horizontal eye images), and the type of analysis (based on stability or distance to the center). By default, the software will calculate phenotypic scores from the first 200 most-ordered ommatidia. The results window can be further downloaded as a .csv file. On an average, the software can process 1 (1800×2400) image every 3 seconds.

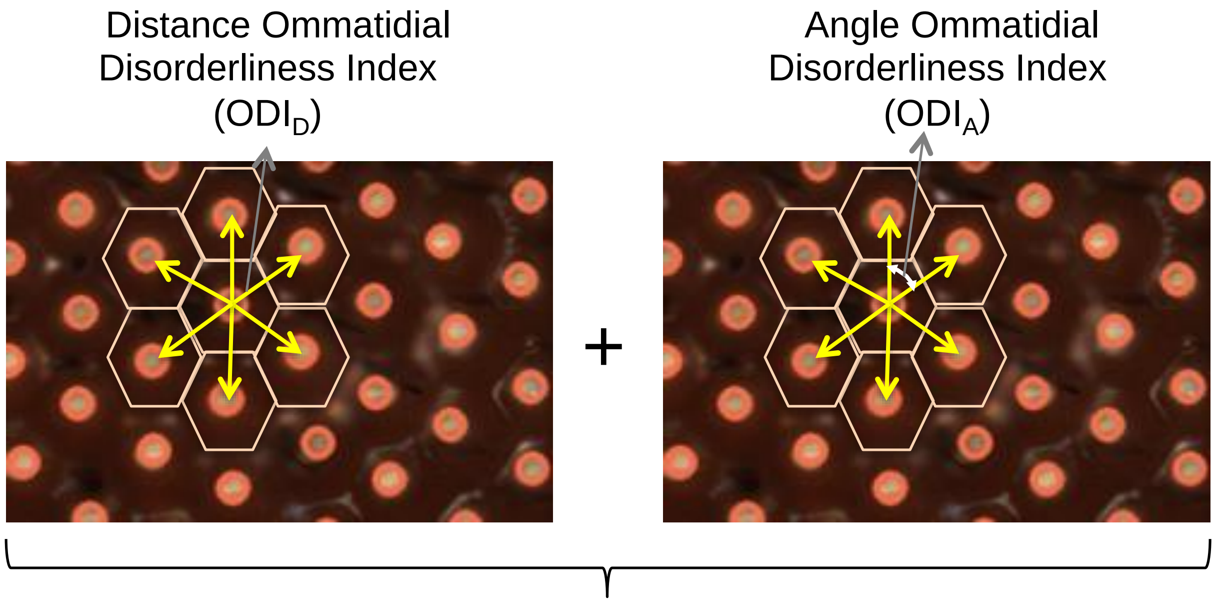

Total Ommatidial Disorderliness Index, $ODI_T = ODI_D + ODI_A$

Phenotypic score, $P = (1/\log Z)\ normalized(ODI_T)$

Figure S4. Calculation of phenotypic score.
The figure shows a part of the fly eye with seven ommatidia shown as hexagons in white and the center of ommatidia identified as smaller orange circles within these hexagons. Four metrics are used for the calculation of phenotypic score (ODI_D, ODI_A, ODI_T and fusion index). Six local vectors with direction pointing from each ommatidium to the neighboring ommatidia are shown (yellow arrows). The distance ommatidial disorderliness index, ODI_D, is calculated as the difference between the lengths of each of the five local vectors from the smallest vector. The angle ommatidial disorderliness index, ODI_A, is measured as the difference between the angles formed between pairs of the five local vectors to the smallest angle. The total ommatidial disorderliness index, ODI_T, is the sum of distance and angle ommatidial disorderliness indices using the number of most ordered ommatidia. See Methods and formula (3). Fusion index is the number of ommatidia detected. The sum of the two entropy (disorderliness) measures (ODI_T calculated from ODI_D and ODI_A) and fusion index measures (Z) are used to calculate the phenotypic score using the depicted formula.

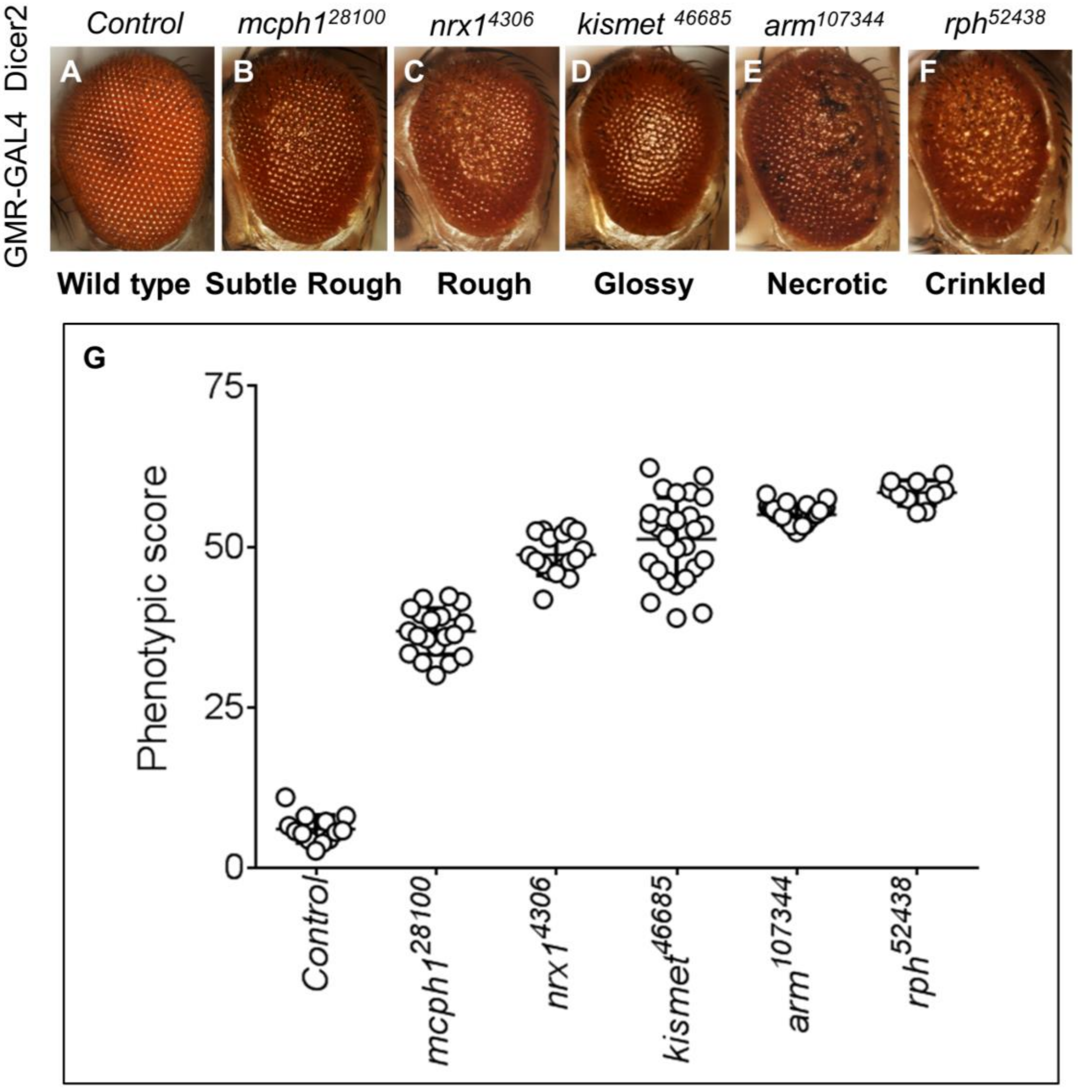

Figure S5. Analysis of different categories of eye phenotypes.
(**A-F**) Representative bright-field microscope eye images displaying eye-specific knockdown of *mcph1*, *nrx-1*, *kismet*, *arm*, and *rph* genes from flies reared at 30°C. Eyes of GMR-GAL4; Dicer2/+ control flies show normal ommatidial organization, while the eyes of flies with GMR-GAL4 driven RNAi knockdown of the 5 genes show disruption in the morphology of the eye that can be classified into different categories. (**G**) Graph representing the phenotypic scores of control flies compared to 5 different categories of eye morphology. The number of images analyzed for each of these genotypes is as follows: control (n=22), *mcph1* (n=20), *nrx-1* (n=17), *kismet* (n=27), *arm* (n=25), and *rph* (n=10).

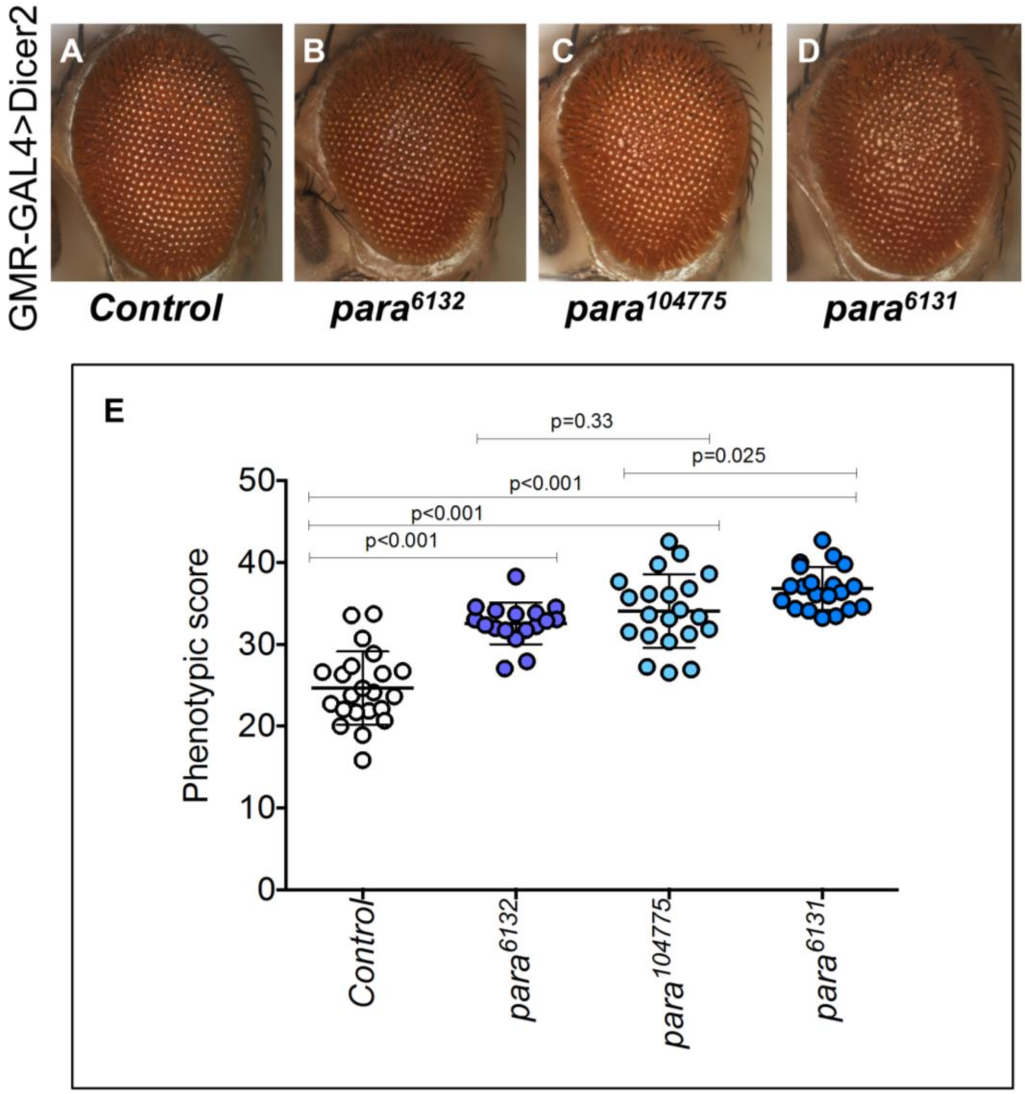

Figure S6. Phenotypic analysis of fly lines with RNAi mediated knockdown of para (*SCN1A* in humans).

(**A-D**) Representative bright-field microscope images of fly eyes displaying eye specific knockdown (using GMR-GAL4) of *para* in three different RNAi lines, $para^{6131}$, $para^{6132}$ and $para^{104775}$ are shown. (**E**) Graphs representing the phenotypic scores of control lines (wVDRC) and three fly lines with *para* knockdown are also shown. Phenotypic scores of these three RNAi lines enabled us to accurately distinguish between the subtle phenotypic changes. Asterisks (*) represent significant difference by student *t* test (Mann Whitney test, two-tailed p<0.001). The number of images analyzed is as follows: controls (n=22), $para^{6131}$ (n=20), $para^{6132}$ (n=17) and $para^{104775}$ (n=21).

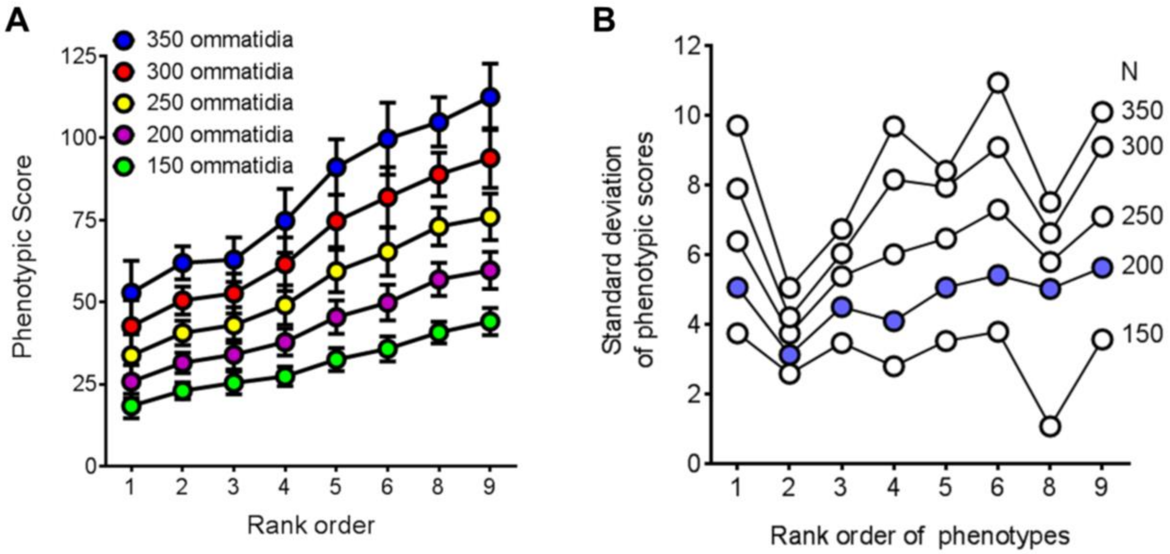

Figure S7. Performance of Flynotyper at different ommatidial counts (N).
(**A**) A graph representing phenotypic scores of genotypes across manually determined ranks (for flies reared at 30°C) for different numbers of ommatidia is shown. The phenotypic scores across the manual ranks ranging from 1 to 9 follow the same trend at different ommatidial counts, reflecting the robustness of our method. (**B**) The variability (denoted by standard deviation) of phenotypic scores at different counts of ommatidia (at N=100, 200, 250, 300, and 350) is shown. The variation is the least for N=200 for genotypes within the same rank or across different rank orders and was adopted as the default parameter in the Flynotyper software for calculation of phenotypic scores. We also note that the user can chose anywhere between 50 and 350, but making sure that this number (N) is smaller than the total number of ommatidia, for calculation of phenotypic scores. For smaller sized eyes, users can choose N as few as 50. N is standard and should be selected only once for all eyes tested in the same experiment. Comparison (or combining) of phenotypic scores across experiments should be performed only if the same number of ommatidia (N) was selected for each of the experiments.

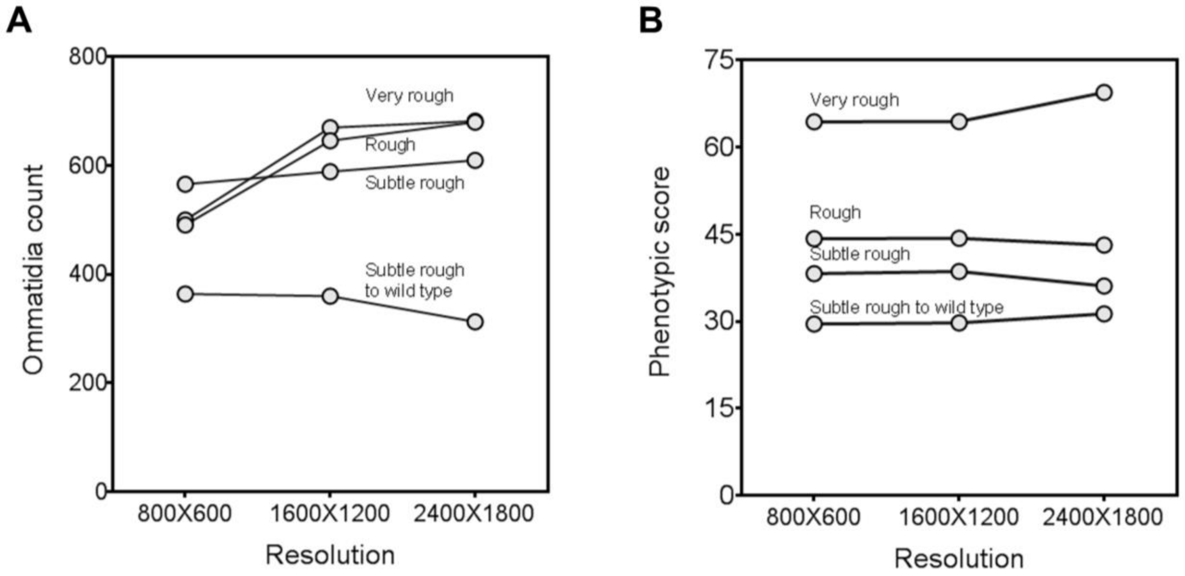

Figure S8. Performance of Flynotyper at image resolutions is shown.
(**A**) Number of ommatidia detected at different image resolutions is shown. (**B**) Phenotypic score calculation at different image resolutions is shown. Note that although the number of ommatidia detected increases with higher resolution images (1200×1600 and 1600×2400), the phenotypic scores are unaltered.

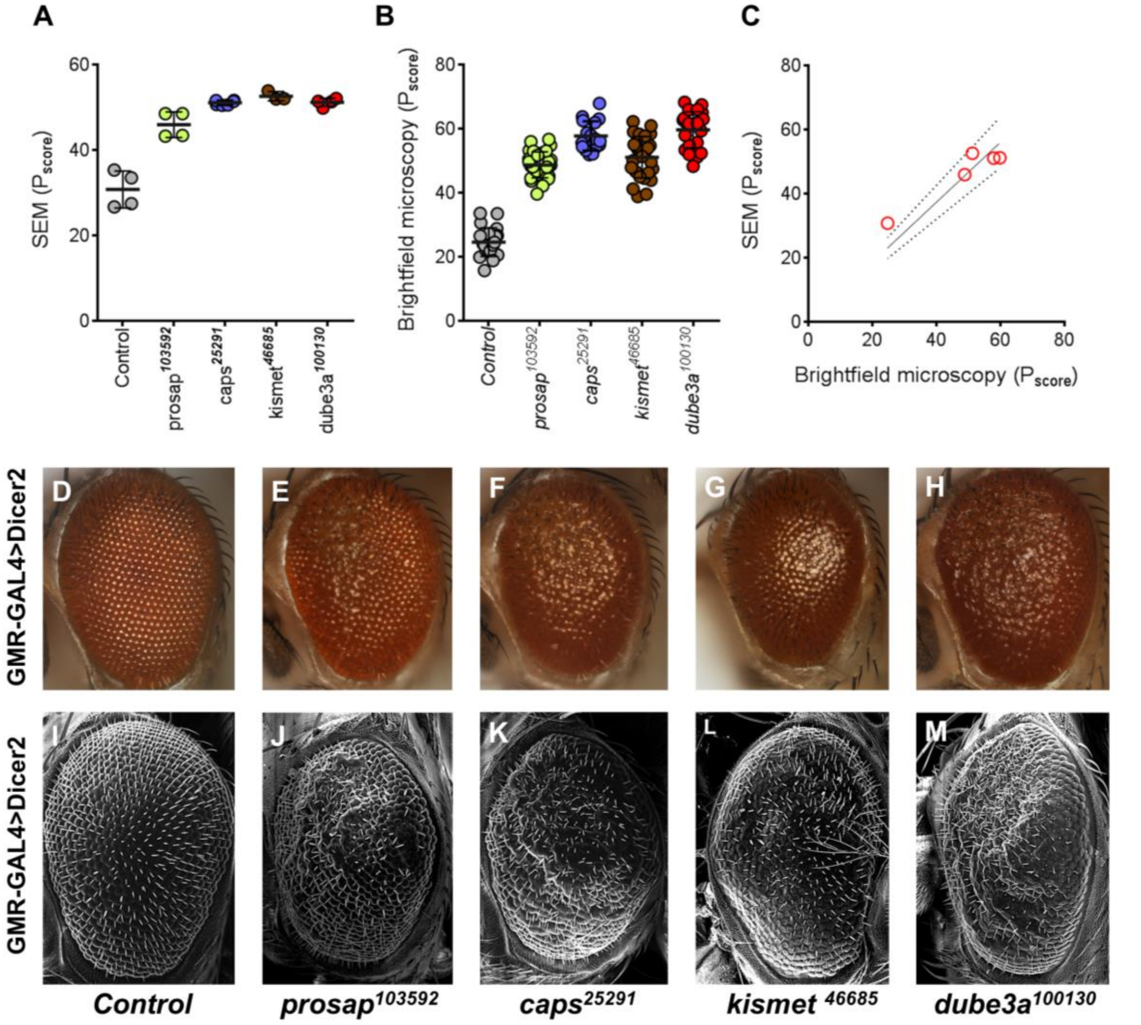

Figure S9. Performance of Flynotyper for SEM and bright field microscope images. Phenotypic scores of GMR-GAL4; Dicer2/+ control flies and fly eyes with GMR-GAL4 driven RNAi knockdown of *prosap*, *caps*, *kismet* and *dube3a* using (**A**) SEM images and (**B**) bright field microscope images are shown. (**C**) A positive correlation is observed (Pearson correlation coefficient to test for linearity, r=0.95, p=0.011) between the scores obtained from SEM compared to bright field microscope. Representative (**D-H**) bright field microscope and corresponding (**I-M**) SEM eye images of GMR-GAL4; Dicer2/+ control flies and fly eyes with RNAi knockdown of *prosap*, *caps*, *kismet* and *dube3a* are also shown. The number of images used for each genotype range from n=4 to n=6 for SEM images and n=7 to n=30 for bright field images.

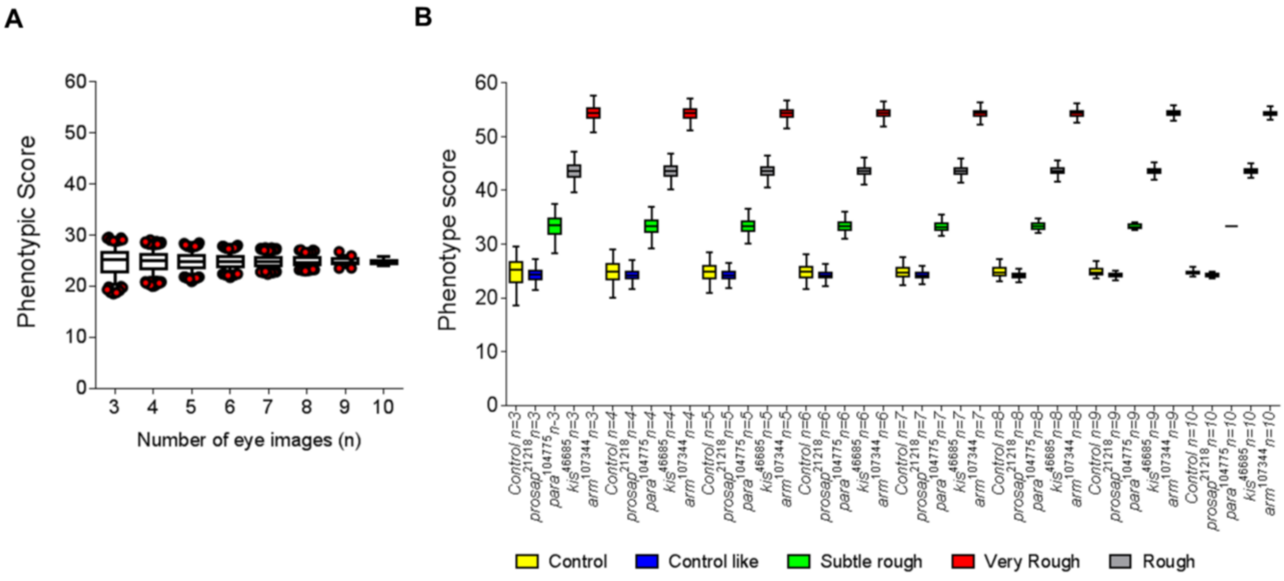

Figure S10. A test for sensitivity of Flynotyper and its ability to distinguish between different classes of phenotypes.

(**A**) The plot shows the phenotypic scores when combinations ($^{10}C_3$ number of combinations) of eye images at n=3 to n=10 are tested. Each box plot shows the distribution of mean phenotypic score (mean and SD) for each combination of eye images. Note that the distribution is tighter for larger numbers of eye images. (**B**) Distribution of mean phenotypic scores for each combination of n=3 to n=10 images were tested. Each of the fly eye phenotypic categories was tested against each other using a Mann Whitney test. While no difference in the distribution of mean phenotypic scores (for n=3 to n=10) was observed between control and control like eye images, significant differences were observed for each of the other categories compared to each other and with the control eye images (two-tailed $p<0.001$, Mann Whitney test).

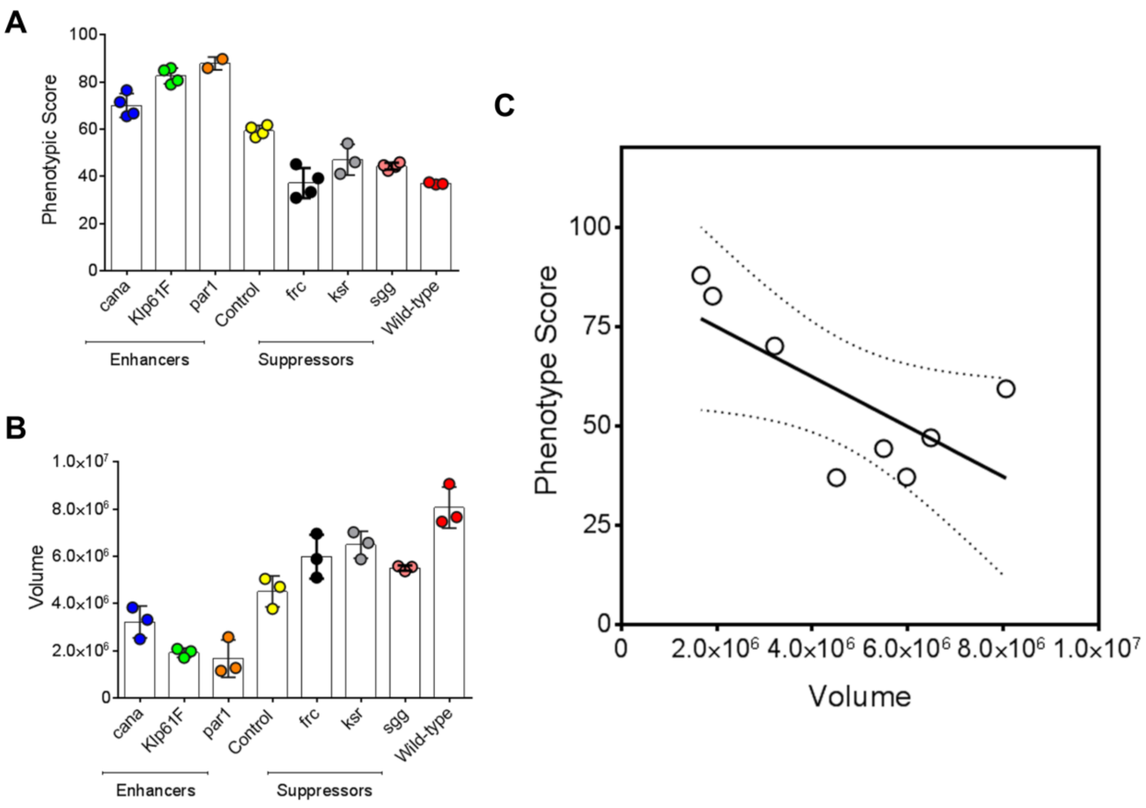

Figure S11. Validation of Flynotyper for images obtained from independent studies (Example 1).

Example of validation using data from Ambegaokar and Jackson (Ambegaokar and Jackson 2011). **(A)** A graph representing the phenotypic scores of wild-type, control, and the three enhancers and suppressors of w^{1118}/+;*gl-tau*/+ is shown. The control listed is w^{1118}/+;*gl-tau*/+. All other panels, except wld-type, contain one copy of *gl-tau* transgene *in trans* to one disrupted copy of the gene listed in the panel. **(B)** A graph representing the eye volumes of wild-type, control, and the three enhancers and suppressors of w^{1118}/+;*gl-tau*/+ is shown. **(C)** Correlation plot of phenotypic scores and eye volumes shown significantly negative correlation between the volume of the eyes and the phenotypic scores (Pearson r=-0.71, two-tailed p=0.049). These observations suggest that the phenotypic scores for the modifiers are concordant with the original assessment. The number of images used for these analyses were n=2 for par1, n=3 each for wild type and ksr, n=4 each for control, cana, Klp61F, sgg, and frc.

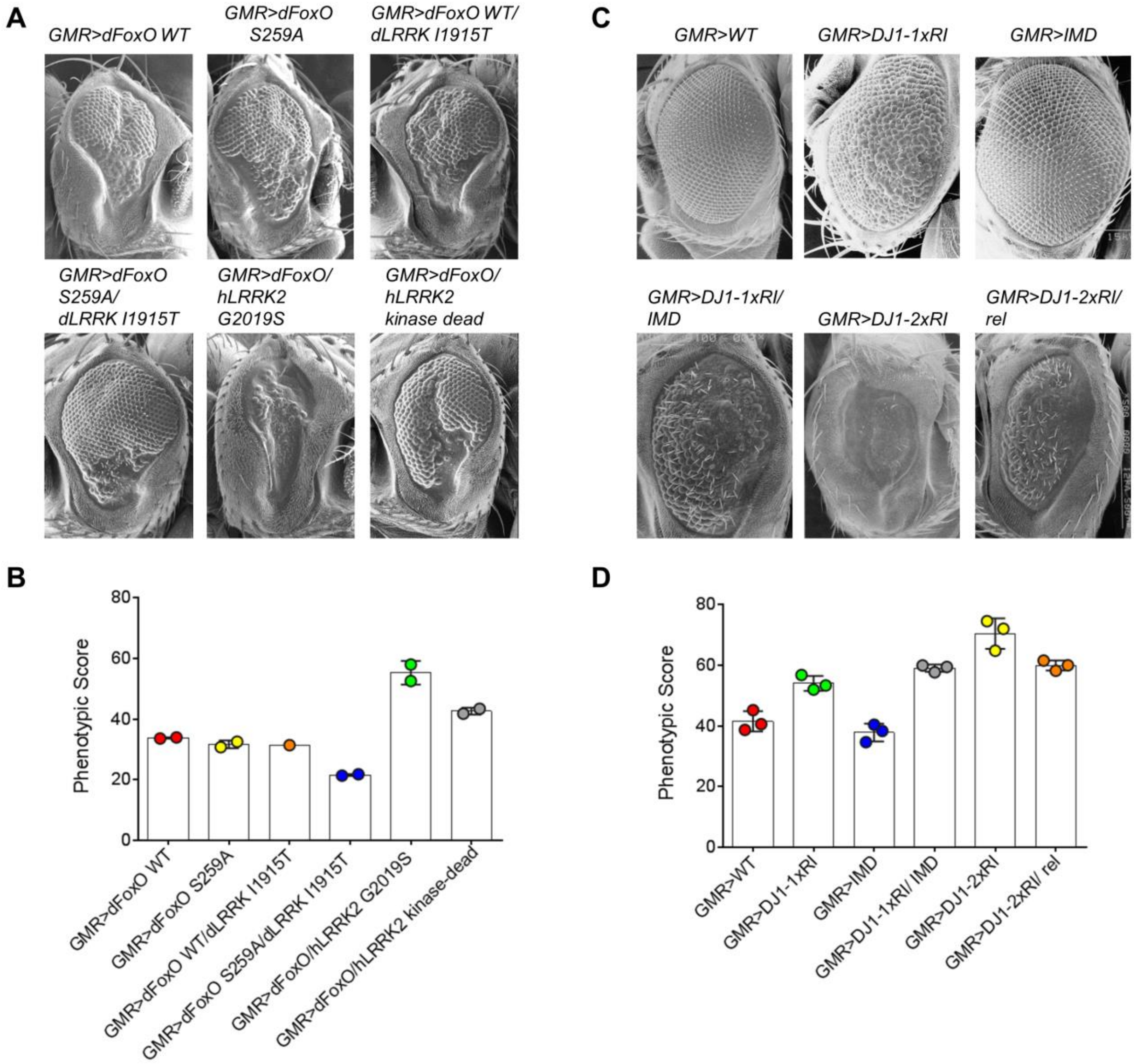

Figure S12. Validation of Flynotyper for images obtained from independent studies (Example 2).

(A) High resolution SEM images of interactors of *dFoxO*. While, the eye degeneration phenotype of *dFoxO S259A* mutation is partially rescued by *dLRRK I1915T*, *hLRRK2 G2019S and hLRRK2 kinase dead* mutations enhance the eye phenotype caused by expression of *dFoxO*. **(B)** A graph representing the phenotypic scores of interactors of *dFoxO* using the GMR-GAL4 driver is shown. The phenotypic scores are concordant with the visual assessment of the eye phenotypes, showing that *LRRK* and *dFoxO* are interactors. The number of images processed were n=1 for two genotypes and n=2 for the remaining six genotypes. **(C)** SEM images of RNAi knockdown of *DJ-1* and its modifiers. While IMD over expression by itself has no effect on fly eye morphology, it enhanced the rough-eye phenotype caused by moderate *DJ-1* RNAi (*DJ1-*

1xRI: one copy of the *DJ-1* RNAi transgene driven by GMR-Gal4). On the other hand, loss of one copy of *relish* (*rel*/+), a key component in the IMD pathway, partially rescued the strong eye phenotype caused by over expression of 2 copies of the *DJ-1* RNAi transgene (*DJ1-2xRI*). (**D**) A graph representing the phenotypic scores of modifiers of *DJ-1* RNAi using the GMR-GAL4 driver is shown. The phenotypic scores are concordant with the visual assessment of the eye phenotypes and suggest that *IMD* and *rel* interact with *DJ-1*. The number of images processed were n=3 for all the samples.

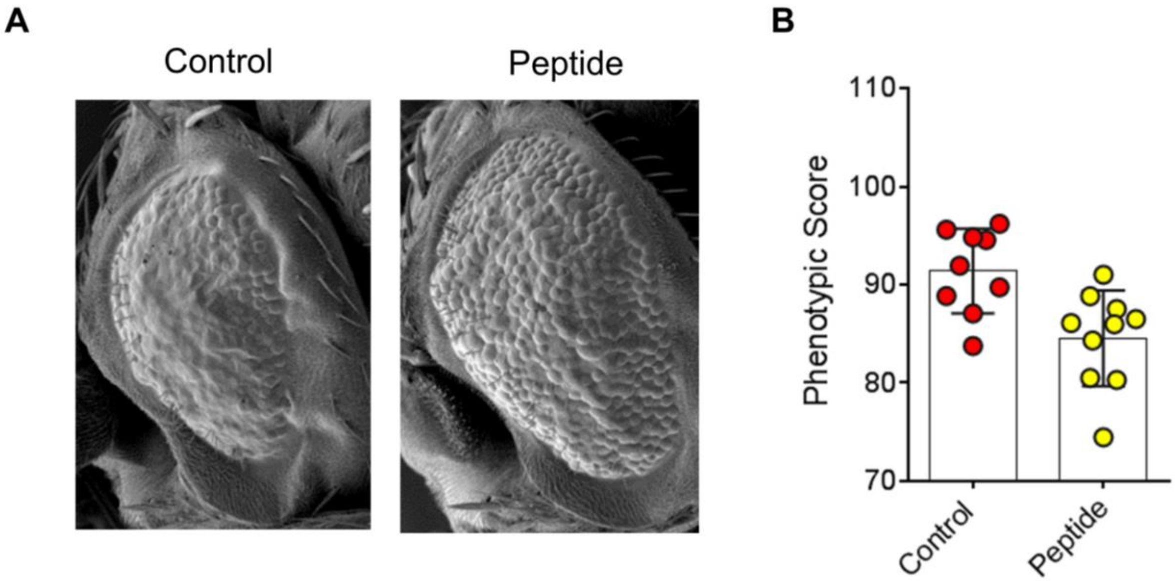

Figure S13. Suppression of UAS-i(CTG)480 toxicity by transgenic expression of ABP1 peptide.
(A) Representative SEM eye images of control (GMR-Gal4>*UAS-i(CTG)$_{480}$UAS-GFP*) and peptide (GMR-Gal4>*UAS-i(CTG)$_{480}$UAS-ABP1*). Expression of *UAS-i(CTG)$_{480}$* caused roughness and reduced eye size. This was significantly suppressed with *UAS-ABP1* peptide expression. **(B)** A graph representing the phenotypic scores of control and peptide treatment. The phenotypic scores are concordant with the visual assessment and show that the ABP1 peptide treatment suppresses the eye phenotype caused by expression of *UAS-i(CTG)$_{480.}$* The number of images processed were n=9 for controls and n=10 for peptide treated eyes.

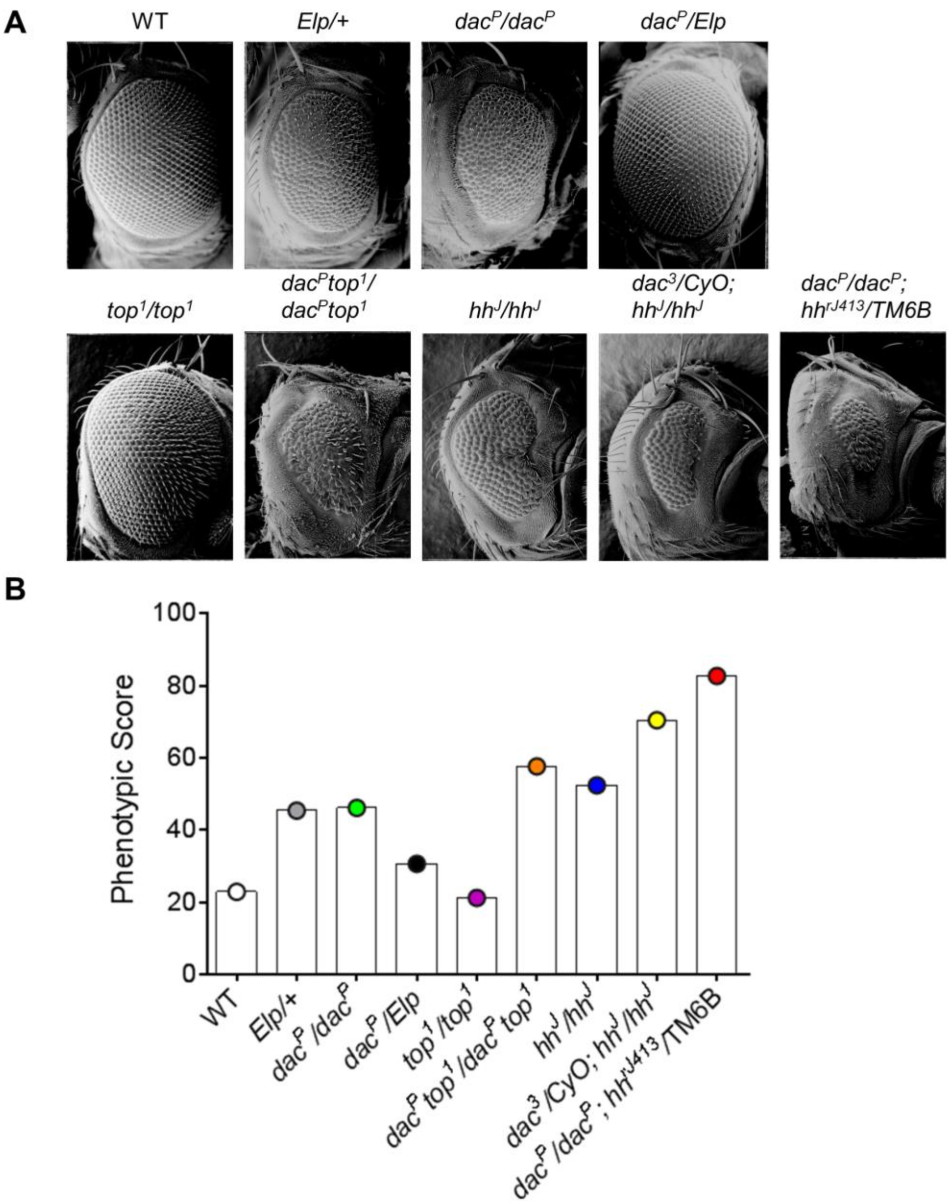

Figure S14. A genetic screen for interactors of Egfr using P-element insertions.
(**A**) High resolution SEM images of interactors of *Egfr* and *dac*. While *Elp* (a dominant allele of *Egfr*) heterozygote that has rough eyes, is suppressed by a single copy of dac^{P}, top^{1}, a weak allele of *Egfr*, enhances the dac^{P} rough eye phenotype. Similarly, the dac^{P} rough eye phenotype is enhanced *by* hh^{rJ413} allele of hedgehog gene. Additionally, hh^{J} allele enhances dac^{3} rough eye phenotype. These results indicate interaction between *Egfr* and *dac* and also *dac* and *hh*. (**B**) A graph representing the phenotypic scores of the interactors of *Egfr* and *dac*. The phenotypic scores also confirm the interaction between *Egfr* and *dac* and also *dac* and *hh.*

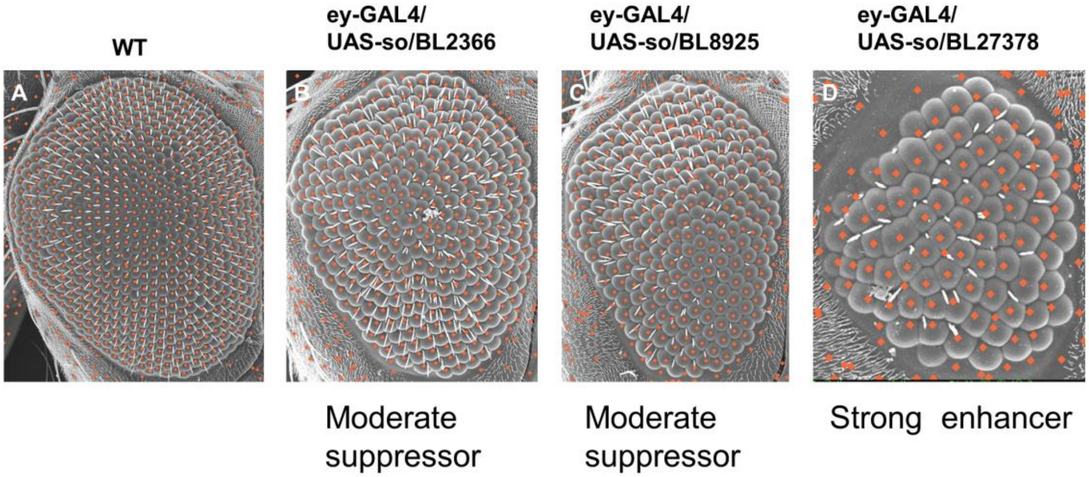

Figure S15. Flynotyper analysis of SEM images to identify modifiers of *sine oculis*. Representative SEM eye images of wild type **(A)** and genetic modifiers of *sine oculis* (*so*) (**B**-**D**), displaying their ommatidial centers (red dots) as detected by Flynotyper are shown.

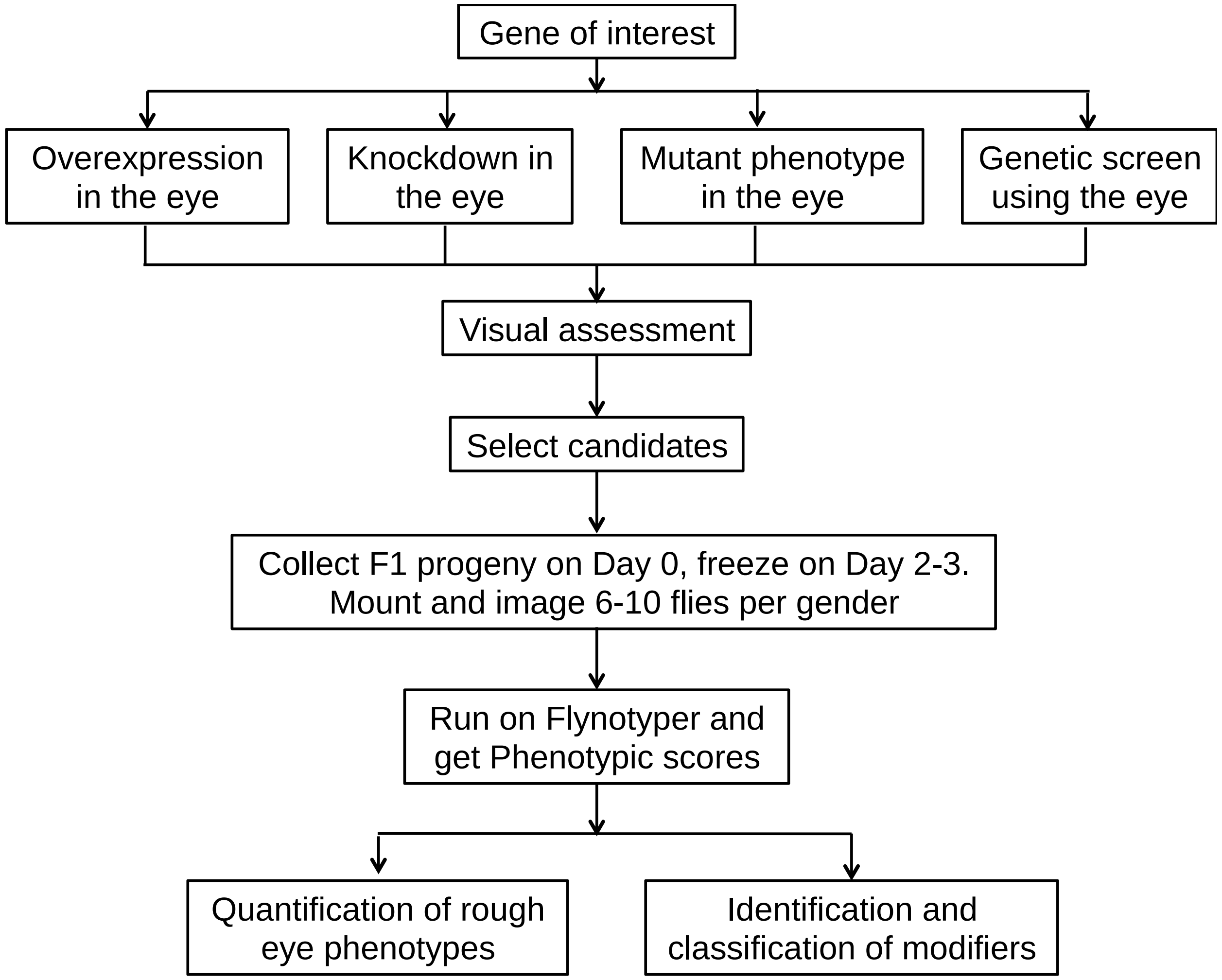

Figure S16. Flowchart depicting the utility of Flynotyper.
A schematic for the proposed use of Flynotyper is shown. Flynotyper can be used for understanding the effects of genetic mutations, gene-dosage alteration, and modifier genes. For genome-wide modifier screens, usually visual inspection under the microscopy is used to identify a subset of candidates that is further classified as enhancers and suppressors using Flynotyper. The method is automated in that the images do not need to be delineated for the region of interest. The Flynotyper software will automatically detect the fly eye and process each image for phenotype score.

Supplementary Tables

Table S1. Stock list of candidate genotypes prioritized from the deficiency screen of modifiers of UAS-*so*

Stock #	Genotype
BL442	Df(2R)CX1, wg12 b1 pr1/SM1
BL727	Df(1)g, f[1] B[1]/In(1)AM
BL1931	Df(3R)by10, red1 e1/TM3, Sb1 Ser1
BL2366	Df(3R)XTA1, Diap11 st1 kniri-1 rnroe-1 p1/Dp(3;3)M95A+13, st1 e1
BL2414	Df(2L)spdj2, wgspd-j2/CyO, P{ftz/lacB}E3
BL3520	wa Nfa-g; Df(2R)Jp8, w+/CyO
BL3347	Df(1)sd72b/FM7c
BL7144	Df(2L)BSC37, dpp[EP2232]/CyO
BL7659	w1118; Df(3R)Exel6180, P{XP-U}Exel6180/TM6B, Tb1
BL7689	w1118; Df(3R)Exel6211, P{XP-U}Exel6211/TM6B, Tb1
BL8674	w1118; Df(2L)BSC109/CyO
BL8925	w1118; Df(3R)ED6316, P{3'.RS5+3.3'}ED6316/TM6C, cu1 Sb1
BL18322	w1118; PBac{WH}Cpsf100f00376/TM6B, Tb1
BL25005	w1118; Df(3R)BSC501/TM6C, Sb1 cu1
BL27378	w1118; Df(3R)BSC806, P+PBac{XP3.RB5}BSC806/TM6C, Sb1 cu1
BL34665	y1 sc* v1; P{TRiP.HMS01142}attP2

Table S2. Primers used for quantitative real time PCR

**Primers**	**Sequence**
para (forward)	5' AGCTATACCGTCCATCTTCAATG 3'
para (reverse)	5' TTTGGTATGATCTCGTGGCTG 3'
prosap (forward)	5' CCGGAGCTGAATGTCTACAAG 3'
prosap (reverse)	5' GCAAACAGGCCATAGTTGAAG 3'
kismet (forward)	5' AAGACGTTCATCTGGGACTTG 3'
kismet (reverse)	5' GTTTGACTTTCCACCGTTGC 3'
caps (forward)	5' TCCCACAAACCAACTCCAC 3'
caps (reverse)	5' CAAAAGTACAAGGATCGGTGC 3'
dube3a (forward)	5' GATTGAAATGCTGGTCTGCG 3'
dube3a (reverse)	5' CACTATGCTCCAGAAGTCCTG 3'
arm (forward)	5' CAGAATCGAACCATGTCGCAT 3'
arm (reverse)	5' CGGCAGATCAGGTGGATTGT 3'
dpten (forward)	5' TCCAATGTTGTAGCCGTGGA 3'
dpten (reverse)	5' GGTACCGGTTCTGCCCTTTC 3'

Table S3. A list of neurodevelopmental genes assessed for eye phenotypes

Human gene (Fly ortholog)	Function	Model organism studies	Phenotypes
SHANK3 (prosap): SH3 and multiple ankyrin repeat domains 3	*SHANK3* is a synaptic scaffolding protein enriched in the postsynaptic density (PSD) of excitatory synapses (Kreienkamp 2008). It plays important roles in synaptogenesis, synaptic plasticity and regulation of dendritic spine morphology (Boeckers et al. 2002).	Mice with *Shank3* haploinsufficiency display defects in synaptic function and plasticity and reduced social interactions (Bozdagi et al. 2010). *Shank3* homozygous mutant mice exhibit abnormal social behavior, repetitive behavior, defects in learning and memory, morphological alterations in dendritic spines, and impaired long term potentiation (Peca et al. 2011; Wang et al. 2011)	ASD (Moessner et al. 2007; Gauthier et al. 2009), language and/or social communication impairment (Durand et al. 2007).
UBE3A (dube3a): Ubiquitin protein ligase E3A	UBE3A is part of the ubiquitin protein degradation system, which accepts ubiquitin from an E2 ubiquitin-conjugating enzyme in the form of a thioester and transfers it to its substrates (Hershko and Ciechanover 1998)	*Ube3A* maternal-deficient mice display reduced brain weight, ataxia, motor defects and behavioral defects (Jiang et al. 1998; Heck et al. 2008). Lack of *dube3A* in *Drosophila* leads to motor defects, abnormal circadian rhythms, long-term memory defects and abnormalities in dendritic arborization in the peripheral nervous system (Lu et al. 2009; Wu et al. 2008).	ASD, intellectual disability and developmental delay (Nurmi et al. 2001; Kelleher et al. 2012; Glessner et al. 2009).
SCN1A (para): Sodium Channel, Voltage-Gated, Type I, Alpha Subunit	*SCN1A* encodes a voltage gated sodium channel that is essential for generation and propagation of action potential (Catterall et al. 2010)	Mice with a loss-of-function nonsense mutation in *Scn1a* develop epileptic seizures within the first postnatal month (Ogiwara et al. 2007). Mice with *Scn1a* haploinsufficiency exhibit hyperactivity, stereotyped behaviors, social interaction deficits and impaired context-dependent	Dravet's syndrome (childhood neuropsychiatric disorder including intractable seizures, cognitive deficits and ASD) (Claes et al. 2001;

		spatial memory and these features are rescued by a low-dose clonazepam, a positive allosteric modulator of GABA(A) receptors (Han et al. 2012).	Frosk et al. 2013), epilepsy (Epi et al. 2013; Kasperaviciute et al. 2013) and ASD (Weiss et al. 2003; O'Roak et al. 2011).
PTEN (dpten): Phosphatase and Tensin Homolog	*PTEN* is a tumor suppressor with lipid phosphatase activity (PMID: 10564676).It is a protein phosphatase, that can dephosphorylate both serine and threonine residues. It plays an important role in PI3 kinase/AKT pathway, MAPK pathway and in the mediation of growth arrest (Ali et al. 1999).	*Pten* haploinsufficient mice exhibit social defects and repetitive behavior (PMID: 22900024). Mice with CNS deletion of Pten displayed abnormal social behavior and defects in social interactions (Waite and Eng 2002)	Cancer (Napoli et al. 2012; Kwon et al. 2006), Macrocephaly, autism (Waite and Eng 2002; Goffin et al. 2001; O'Roak et al. 2012a), learning disabilities (Busa et al. 2013) and epilepsy (Epi et al. 2013).
CADPS2 (caps): Calcium dependent activator protein for secretion	*CADPS2* regulates exocytosis of dense core vesicles (Grishanin et al. 2004)	*Cadps2* knockout mice display impaired cerebellar development and function and autistic-like cellular and behavioral phenotypes (Sadakata et al. 2007)	Autism (Cisternas et al. 2003; Okamoto et al. 2011; Girirajan et al. 2013), Intellectual disability (Bonora et al. 2014).
NRXN1 (nrxn1): Neurexin 1	*Neurexin 1* is a synaptic cell adhesion protein, involved in synaptic formation and function (Chen et al. 2010)	*Nrxn1* deficient mice exhibit defect in excitatory synaptic strength, decrease in prepulse inhibition, an increase in grooming behaviors, an impairment in nest-building activity, and an improvement in motor learning (Etherton et al. 2009).	Autism, intellectual disability, speech delays, seizures, poor muscle tone, unusual facial features (Dabell et al. 2013; Bena et al. 2013).
MCPH1 (mcph1):	*MCPH1* is regulator of chromosome	*Mcph1* null mouse model demonstrates its	Autism (Ozgen et al.

Microcephalin 1	condensation (Trimborn et al. 2004) and is involved in DNA damage induced cellular responses (Xu et al. 2004). May play a role in neurogenesis and regulation of the size of the cerebral cortex (Mahmood et al. 2011).	role in maintaining genomic stability and in regulating programmed and IR-induced DNA damage response (Liang et al. 2010).	2009; Neale et al. 2012), primary microcephaly.
LGR5 (rk): Leucine-rich repeat containing G protein-coupled receptor 5	*LGR5* functions as receptors for R-spondins and regulates canonical Wnt/beta-catenin signaling (Carmon et al. 2011). LGR5 has also been validated as a stem cell marker of the intestinal epithelium (Barker et al. 2007) and the hair follicle (Jaks et al. 2008).	Knockout of *Lgr5* in the mouse leads to total neonatal lethality characterized by gastrointestinal tract dilation and accompanied by ankyloglossia (Morita et al. 2004).	Deletion of human chromosomal region 12q21.1 containing LGR5 is associated with ASD (Thompson et al. 2008).
CHD8 (kismet): Chromodomain-Helicase-DNA-Binding Protein 8	*CHD8* is a DNA helicase that acts as a chromatin-remodeling factor and regulates transcription. Acts as a negative regulator of Wnt signaling pathway by regulating beta-catenin activity (Thompson et al. 2008).	*Chd8* null mice die during early embryogenesis due to widespread apoptosis. CHD8 plays a role in regulating tumor suppressor p53 activity and prevents apoptosis mediated by p53 (Nishiyama et al. 2009).	Autism, gastrointestinal disorders, macrocephaly and characteristic facial features, including wide-set eyes, large ears, broad foreheads and noses (Bernier et al. 2014).
CTNNB1 (arm): Catenin (cadherin-associated protein), beta 1	*CTNNB1* is a pivotal component of the canonical Wnt signaling pathway. Wnt signal stabilizes the beta-catenin, which then accumulates in the cytoplasm and is subsequently translocated to the nucleus, where it	Complete knockout of *Ctnnb1* is embryonic lethal (Haegel et al. 1995). Mutant *Cnnb1* mice with deletion restricted to the dorsal telencephalon, surivive to adulthood and show increased susceptibility to seizures and cortical malformation (Campos et al.	Autism, intellectual disability, low muscle tone and microcephaly (Dubruc et al. 2014; Tucci et al. 2014; de Ligt et al. 2012; O'Roak et al.

	interacts with members of the TCF family of transcription factors and induces the transcription of Wnt target genes (Willert and Nusse 1998).	2004)	2012b)
EPHA6 (eph): Ephrin Type-A Receptor 6	*EPHA6* is a member of Eph family of tyrosine kinases that are involved in development of neuronal projection pathways (Martinez and Soriano 2005)	*EphA6* knockout mice exhibit learning and memory impairments (Savelieva et al. 2008)	ASD (Girirajan et al. 2013; Pinto et al. 2010).
SLC25A19 (tpc1): solute carrier family 25 (mitochondrial thiamine pyrophosphate carrier), member 19	*SLC25A19* is involved in the transport of thiamine pyrophosphate (Kang and Samuels 2008)	*Slc25a19* knockout mice display mitochondrial pyrophosphate depletion, embryonic lethality, CNS malformations and anemia (Lindhurst et al. 2006).	Amish lethal microcephaly (Rosenberg et al. 2002), bilateral striatal necrosis and chronic progressive polyneuropathy (Spiegel et al. 2009).

Table S4. ***Drosophila*** **orthologs of human neurodevelopmental genes and the qualitative rank order of their eye phenotypes**

Number	Human Gene	*Drosophila* ortholog	Stock#	Rank order
1	*SHANK3*	*prosap*	21218	1
2	*LGR5*	*rk*	105360	1
3	*SCN1A*	*para*	6132	2
4	*UBE3A*	*dube3a*	45876	2
5	*EPHA6*	*eph*	6545	2
6	*LGR5*	*rk*	29932	2
7	*SCN1A*	*para*	104775	3
8	*SLC25A19*	*tpc1*	6005	3
9	*SCN1A*	*para*	6131	3.5
10	*LGR5*	*rk*	29931	4
11	*MCPH1*	*mcph1*	28100	4
12	*PTEN*	*dpten*	101475	4
13	*CADPS2*	*caps*	25292	5
14	*CHD8*	*kismet*	46685	6
15	*SHANK3*	*prosap*	103592	6.5
16	*NRXN-1*	*nrx-1*	4306	7
17	*MCPH1*	*mcph1*	106261	8
18	*CTNNB1*	*arm*	107344	8
19	*CADPS2*	*caps*	25291	8.5
20	*UBE3A*	*dube3a*	100130	9
21	*PTEN*	*dpten*	35731	9

Table S5. Student *t* test comparing eye phenotypes of neurodevelopmental genes with controls

Genotypes	Number of samples	Two-tailed p value	Two-tailed p value (After multiple testing correction)
Control	22	1	-
$prosap^{21218}$	18	0.498	-
rk^{105360}	22	3.93×10^{-04}	8.25×10^{-03}
$dube3a^{45876}$	25	4.06×10^{-06}	8.52×10^{-05}
eph^{6545}	22	1.97×10^{-06}	4.14×10^{-05}
rk^{29932}	12	3.28×10^{-06}	6.89×10^{-05}
$para^{6132}$	17	4.76×10^{-08}	9.99×10^{-07}
$para^{104775}$	21	2.49×10^{-08}	5.22×10^{-07}
$tpc1^{6005}$	24	3.67×10^{-11}	7.70×10^{-10}
$para^{6131}$	20	1.11×10^{-12}	2.34×10^{-11}
rk^{29931}	9	4.89×10^{-11}	1.03×10^{-09}
$mcph1^{28100}$	20	4.74×10^{-12}	9.95×10^{-11}
$dpten^{101475}$	18	6.21×10^{-15}	1.30×10^{-13}
$caps^{25292}$	15	3.37×10^{-13}	7.07×10^{-12}
$prosap^{103592}$	30	2.76×10^{-23}	5.79×10^{-22}
$nrx1^{4306}$	17	4.75×10^{-21}	9.98×10^{-20}
$kismet^{46685}$	27	2.17×10^{-21}	4.55×10^{-20}
$mcph1^{106261}$	21	1.46×10^{-24}	3.07×10^{-23}
arm^{107344}	25	2.22×10^{-21}	4.66×10^{-20}
$caps^{25291}$	17	3.82×10^{-22}	8.03×10^{-21}
$dpten^{35731}$	17	4.55×10^{-15}	9.55×10^{-14}
$dube3a^{100130}$	24	3.23×10^{-26}	6.79×10^{-25}

Multiple testing corrections were performed using Bonferroni method

Table S6. Student *t* test comparing phenotypic scores of eye phenotypes at 28°C to that at 30°C

Fly genotype	Number of samples (30°C)	Number of samples (28°C)	Student t test, two-tailed p value	Corrected two-tailed p value
Control	22	14	0.009	0.19
prosap^{21218}	18	17	3.81×10^{-07}	6.86×10^{-06}
dube3a^{45876}	25	29	7.71×10^{-19}	1.92×10^{-17}
para^{6131}	17	24	8.98×10^{-16}	1.52×10^{-14}
para^{6132}	20	23	4.25×10^{-19}	8.50×10^{-18}
dpten^{101475}	18	23	1.29×10^{-11}	2.33×10^{-10}
caps^{25292}	15	24	5.74×10^{-07}	8.61×10^{-06}
prosap^{103592}	30	26	3.84×10^{-28}	1.15×10^{-26}
arm^{107344}	27	24	1.51×10^{-19}	4.09×10^{-18}
caps^{25291}	25	17	3.77×10^{-05}	0.00094
dpten^{35731}	17	20	8.96×10^{-10}	1.52×10^{-08}
kismet^{46685}	17	20	3.60×10^{-12}	6.12×10^{-11}
dube3a^{100130}	24	25	6.64×10^{-26}	1.59×10^{-24}

Student t test was used to calculate two tailed p values. Multiple-testing correction was performed using Bonferroni method.

Table S7. Student *t* test comparing the phenotypic scores of modifiers of UAS-*so* with phenotypic scores from UAS-*so* alone

Genotype	Number of SEM images	One-tailed p value*	Corrected one-tailed p value[a]	Two-tailed p value*	Corrected two-tailed p value[a]	Modifier
eyG4_UAS-so_BL27378	2	0.1774	-	0.35	-	Strong enhancer
eyG4_UAS-so_BL7689	2	0.1078	-	0.21	-	Strong enhancer
eyG4_UAS-so_BL18322	2	0.0855	0.76	0.17	-	Moderate enhancer
eyG4_UAS-so_BL7659	2	0.0531	-	0.10	-	Mild enhancer
**eyG4_UAS-so**	**3**	**0.5000**	**-**	**-**	**-**	**-**
WT	10	-	-	-	-	-
eyG4_UAS-so_BL25005	2	0.2661	-	0.53	-	Mild suppressor
eyG4_UAS-so_BL2366	2	0.0103	0.09	0.02	0.18	Moderate suppressor
eyG4_UAS-so_BL8925	2	0.0086	0.07	0.0173	0.15	Moderate suppressor
eyG4_UAS-so_BL34665	2	0.0069	0.06	0.0137	0.12	Strong suppressor
GMR_UAS-so_BL442	2	0.0247	0.22	0.0495	0.44	Strong enhancer
GMR_UAS-so_BL2414	3	0.0027	0.023	0.0053	0.047	Strong enhancer
GMR_UAS-so_BL1931	2	0.0337	0.3	0.0674	-	Strong enhancer
GMR_UAS-so_BL3520	3	0.3100	-	0.62	-	Mild enhancer
**GMR_UAS-so**	**3**	**0.5000**	**-**		**-**	
GMR_UAS-so_BL7144	3	0.0003	0.0027	0.0006	0.005	Strong suppressor

GMR_UAS-so_BL8674	3	0.0003	0.0026	0.0006	0.005	Strong suppressor
GMR_UAS-so_BL)727	3	0.0002	0.0014	0.0003	0.0029	Strong suppressor
GMR_UAS-so_BL3347	2	0.0040	0.036	0.0081	0.072	Strong suppressor
WT	10	-	-	-	-	-

*Student *t* test was used for calculations. [a]Multiple testing corrections using Bonferroni method were applied.

Table S8. Features and limitations of Flynotyper

**Phenotypes identifiable**	**Flynotyper**
Glossy eye	Yes
Rough eye	Yes
Crinkled eye	Yes
Necrotic eye	Yes
Bristle integrity	No
Ommatidial size	No
Loss of pigmentation	No
Size of the eye	No
**Technical features**	**Flynotyper**
Quantification of eye roughness	Yes
Automatic identification of region of interest	Yes
Requires a manual step prior to processing the images	No
Robust performance with different image acquisition set ups	Yes

References

Ali, I.U., L.M. Schriml, and M. Dean, 1999 Mutational spectra of PTEN/MMAC1 gene: a tumor suppressor with lipid phosphatase activity. *Journal of the National Cancer Institute* 91 (22):1922-1932.

Ambegaokar, S.S., and G.R. Jackson, 2011 Functional genomic screen and network analysis reveal novel modifiers of tauopathy dissociated from tau phosphorylation. *Human Molecular Genetics* 20 (24):4947-4977.

Barker, N., J.H. van Es, J. Kuipers, P. Kujala, M. van den Born *et al.*, 2007 Identification of stem cells in small intestine and colon by marker gene Lgr5. *Nature* 449 (7165):1003-1007.

Bena, F., D.L. Bruno, M. Eriksson, C. van Ravenswaaij-Arts, Z. Stark *et al.*, 2013 Molecular and clinical characterization of 25 individuals with exonic deletions of NRXN1 and comprehensive review of the literature. *American Journal of Medical Genetics. Part B, Neuropsychiatric Genetics* 162B (4):388-403.

Bernier, R., C. Golzio, B. Xiong, H.A. Stessman, B.P. Coe *et al.*, 2014 Disruptive CHD8 mutations define a subtype of autism early in development. *Cell* 158 (2):263-276.

Boeckers, T.M., J. Bockmann, M.R. Kreutz, and E.D. Gundelfinger, 2002 ProSAP/Shank proteins - a family of higher order organizing molecules of the postsynaptic density with an emerging role in human neurological disease. *Journal of Neurochemistry* 81 (5):903-910.

Bonora, E., C. Graziano, F. Minopoli, E. Bacchelli, P. Magini *et al.*, 2014 Maternally inherited genetic variants of CADPS2 are present in autism spectrum disorders and intellectual disability patients. *EMBO Mol Med* 6 (6):795-809.

Bozdagi, O., T. Sakurai, D. Papapetrou, X. Wang, D.L. Dickstein *et al.*, 2010 Haploinsufficiency of the autism-associated Shank3 gene leads to deficits in synaptic function, social interaction, and social communication. *Mol Autism* 1 (1):15.

Busa, T., B. Chabrol, O. Perret, M. Longy, and N. Philip, 2013 Novel PTEN germline mutation in a family with mild phenotype: difficulties in genetic counseling. *Gene* 512 (2):194-197.

Campos, V.E., M. Du, and Y. Li, 2004 Increased seizure susceptibility and cortical malformation in beta-catenin mutant mice. *Biochemical and Biophysical Research Communications* 320 (2):606-614.

Carmon, K.S., X. Gong, Q. Lin, A. Thomas, and Q. Liu, 2011 R-spondins function as ligands of the orphan receptors LGR4 and LGR5 to regulate Wnt/beta-catenin signaling. *Proceedings of the National Academy of Sciences of the United States of America* 108 (28):11452-11457.

Catterall, W.A., F. Kalume, and J.C. Oakley, 2010 NaV1.1 channels and epilepsy. *J Physiol* 588 (Pt 11):1849-1859.

Chen, K., E.O. Gracheva, S.C. Yu, Q. Sheng, J. Richmond *et al.*, 2010 Neurexin in embryonic Drosophila neuromuscular junctions. *PLoS ONE* 5 (6):e11115.

Cisternas, F.A., J.B. Vincent, S.W. Scherer, and P.N. Ray, 2003 Cloning and characterization of human CADPS and CADPS2, new members of the Ca2+-dependent activator for secretion protein family. *Genomics* 81 (3):279-291.

Claes, L., J. Del-Favero, B. Ceulemans, L. Lagae, C. Van Broeckhoven *et al.*, 2001 De novo mutations in the sodium-channel gene SCN1A cause severe myoclonic epilepsy of infancy. *American Journal of Human Genetics* 68 (6):1327-1332.

Dabell, M.P., J.A. Rosenfeld, P. Bader, L.F. Escobar, D. El-Khechen *et al.*, 2013 Investigation of NRXN1 deletions: clinical and molecular characterization. *American Journal of Medical Genetics. Part A* 161A (4):717-731.

de Ligt, J., M.H. Willemsen, B.W. van Bon, T. Kleefstra, H.G. Yntema *et al.*, 2012 Diagnostic Exome Sequencing in Persons with Severe Intellectual Disability. *New England Journal of Medicine* 367 (20):1921-1929.

Dubruc, E., A. Putoux, A. Labalme, C. Rougeot, D. Sanlaville *et al.*, 2014 A new intellectual disability syndrome caused by CTNNB1 haploinsufficiency. *American Journal of Medical Genetics. Part A* 164A (6):1571-1575.

Durand, C.M., C. Betancur, T.M. Boeckers, J. Bockmann, P. Chaste *et al.*, 2007 Mutations in the gene encoding the synaptic scaffolding protein SHANK3 are associated with autism spectrum disorders. *Nature Genetics* 39 (1):25-27.

Epi, K.C., P. Epilepsy Phenome/Genome, A.S. Allen, S.F. Berkovic, P. Cossette *et al.*, 2013 De novo mutations in epileptic encephalopathies. *Nature* 501 (7466):217-221.

Etherton, M.R., C.A. Blaiss, C.M. Powell, and T.C. Sudhof, 2009 Mouse neurexin-1alpha deletion causes correlated electrophysiological and behavioral changes consistent with cognitive impairments. *Proceedings of the National Academy of Sciences of the United States of America* 106 (42):17998-18003.

Frosk, P., A.A. Mhanni, and M.F. Rafay, 2013 SCN1A mutation associated with intractable myoclonic epilepsy and migraine headache. *Journal of Child Neurology* 28 (3):389-391.

Gauthier, J., D. Spiegelman, A. Piton, R.G. Lafreniere, S. Laurent *et al.*, 2009 Novel de novo SHANK3 mutation in autistic patients. *American Journal of Medical Genetics. Part B, Neuropsychiatric Genetics* 150B (3):421-424.

Girirajan, S., M.Y. Dennis, C. Baker, M. Malig, B.P. Coe *et al.*, 2013 Refinement and discovery of new hotspots of copy-number variation associated with autism spectrum disorder. *American Journal of Human Genetics* 92 (2):221-237.

Glessner, J.T., K. Wang, G. Cai, O. Korvatska, C.E. Kim *et al.*, 2009 Autism genome-wide copy number variation reveals ubiquitin and neuronal genes. *Nature* 459 (7246):569-573.

Goffin, A., L.H. Hoefsloot, E. Bosgoed, A. Swillen, and J.P. Fryns, 2001 PTEN mutation in a family with Cowden syndrome and autism. *American Journal of Medical Genetics* 105 (6):521-524.

Grishanin, R.N., J.A. Kowalchyk, V.A. Klenchin, K. Ann, C.A. Earles *et al.*, 2004 CAPS acts at a prefusion step in dense-core vesicle exocytosis as a PIP2 binding protein. *Neuron* 43 (4):551-562.

Haegel, H., L. Larue, M. Ohsugi, L. Fedorov, K. Herrenknecht *et al.*, 1995 Lack of beta-catenin affects mouse development at gastrulation. *Development* 121 (11):3529-3537.

Han, S., C. Tai, R.E. Westenbroek, F.H. Yu, C.S. Cheah *et al.*, 2012 Autistic-like behaviour in Scn1a+/- mice and rescue by enhanced GABA-mediated neurotransmission. *Nature* 489 (7416):385-390.

Heck, D.H., Y. Zhao, S. Roy, M.S. LeDoux, and L.T. Reiter, 2008 Analysis of cerebellar function in Ube3a-deficient mice reveals novel genotype-specific behaviors. *Human Molecular Genetics* 17 (14):2181-2189.

Hershko, A., and A. Ciechanover, 1998 The ubiquitin system. *Annual Review of Biochemistry* 67:425-479.

Jaks, V., N. Barker, M. Kasper, J.H. van Es, H.J. Snippert *et al.*, 2008 Lgr5 marks cycling, yet long-lived, hair follicle stem cells. *Nature Genetics* 40 (11):1291-1299.

Jiang, Y.H., D. Armstrong, U. Albrecht, C.M. Atkins, J.L. Noebels *et al.*, 1998 Mutation of the Angelman ubiquitin ligase in mice causes increased cytoplasmic p53 and deficits of contextual learning and long-term potentiation. *Neuron* 21 (4):799-811.

Kang, J., and D.C. Samuels, 2008 The evidence that the DNC (SLC25A19) is not the mitochondrial deoxyribonucleotide carrier. *Mitochondrion* 8 (2):103-108.

Kasperaviciute, D., C.B. Catarino, M. Matarin, C. Leu, J. Novy *et al.*, 2013 Epilepsy, hippocampal sclerosis and febrile seizures linked by common genetic variation around SCN1A. *Brain* 136 (Pt 10):3140-3150.

Kelleher, R.J., 3rd, U. Geigenmuller, H. Hovhannisyan, E. Trautman, R. Pinard *et al.*, 2012 High-throughput sequencing of mGluR signaling pathway genes reveals enrichment of rare variants in autism. *PLoS ONE* 7 (4):e35003.

Kreienkamp, H.J., 2008 Scaffolding proteins at the postsynaptic density: shank as the architectural framework. *Handb Exp Pharmacol* (186):365-380.

Kwon, C.H., B.W. Luikart, C.M. Powell, J. Zhou, S.A. Matheny *et al.*, 2006 Pten regulates neuronal arborization and social interaction in mice. *Neuron* 50 (3):377-388.

Liang, Y., H. Gao, S.Y. Lin, G. Peng, X. Huang *et al.*, 2010 BRIT1/MCPH1 is essential for mitotic and meiotic recombination DNA repair and maintaining genomic stability in mice. *PLoS Genetics* 6 (1):e1000826.

Lindhurst, M.J., G. Fiermonte, S. Song, E. Struys, F. De Leonardis *et al.*, 2006 Knockout of Slc25a19 causes mitochondrial thiamine pyrophosphate depletion, embryonic lethality, CNS malformations, and anemia. *Proceedings of the National Academy of Sciences of the United States of America* 103 (43):15927-15932.

Lu, Y., F. Wang, Y. Li, J. Ferris, J.A. Lee *et al.*, 2009 The Drosophila homologue of the Angelman syndrome ubiquitin ligase regulates the formation of terminal dendritic branches. *Human Molecular Genetics* 18 (3):454-462.

Mahmood, S., W. Ahmad, and M.J. Hassan, 2011 Autosomal Recessive Primary Microcephaly (MCPH): clinical manifestations, genetic heterogeneity and mutation continuum. *Orphanet Journal of Rare Diseases* 6:39.

Martinez, A., and E. Soriano, 2005 Functions of ephrin/Eph interactions in the development of the nervous system: emphasis on the hippocampal system. *Brain Research. Brain Research Reviews* 49 (2):211-226.

Moessner, R., C.R. Marshall, J.S. Sutcliffe, J. Skaug, D. Pinto *et al.*, 2007 Contribution of SHANK3 mutations to autism spectrum disorder. *American Journal of Human Genetics* 81 (6):1289-1297.

Morita, H., S. Mazerbourg, D.M. Bouley, C.W. Luo, K. Kawamura *et al.*, 2004 Neonatal lethality of LGR5 null mice is associated with ankyloglossia and gastrointestinal distension. *Molecular and Cellular Biology* 24 (22):9736-9743.

Napoli, E., C. Ross-Inta, S. Wong, C. Hung, Y. Fujisawa *et al.*, 2012 Mitochondrial dysfunction in Pten haplo-insufficient mice with social deficits and repetitive behavior: interplay between Pten and p53. *PLoS ONE* 7 (8):e42504.

Neale, B.M., Y. Kou, L. Liu, A. Ma'ayan, K.E. Samocha *et al.*, 2012 Patterns and rates of exonic de novo mutations in autism spectrum disorders. *Nature* 485 (7397):242-245.

Nishiyama, M., K. Oshikawa, Y. Tsukada, T. Nakagawa, S. Iemura *et al.*, 2009 CHD8 suppresses p53-mediated apoptosis through histone H1 recruitment during early embryogenesis. *Nature Cell Biology* 11 (2):172-182.

Nurmi, E.L., Y. Bradford, Y. Chen, J. Hall, B. Arnone *et al.*, 2001 Linkage disequilibrium at the Angelman syndrome gene UBE3A in autism families. *Genomics* 77 (1-2):105-113.

O'Roak, B.J., P. Deriziotis, C. Lee, L. Vives, J.J. Schwartz *et al.*, 2011 Exome sequencing in sporadic autism spectrum disorders identifies severe de novo mutations. *Nature Genetics* 43 (6):585-589.

O'Roak, B.J., L. Vives, W. Fu, J.D. Egertson, I.B. Stanaway *et al.*, 2012a Multiplex targeted sequencing identifies recurrently mutated genes in autism spectrum disorders. *Science* 338 (6114):1619-1622.

O'Roak, B.J., L. Vives, S. Girirajan, E. Karakoc, N. Krumm *et al.*, 2012b Sporadic autism exomes reveal a highly interconnected protein network of de novo mutations. *Nature* 485 (7397):246-250.

Ogiwara, I., H. Miyamoto, N. Morita, N. Atapour, E. Mazaki *et al.*, 2007 Nav1.1 localizes to axons of parvalbumin-positive inhibitory interneurons: a circuit basis for epileptic seizures in mice carrying an Scn1a gene mutation. *Journal of Neuroscience* 27 (22):5903-5914.

Okamoto, N., Y. Hatsukawa, K. Shimojima, and T. Yamamoto, 2011 Submicroscopic deletion in 7q31 encompassing CADPS2 and TSPAN12 in a child with autism spectrum disorder and PHPV. *American Journal of Medical Genetics. Part A* 155A (7):1568-1573.

Ozgen, H.M., E. van Daalen, P.F. Bolton, V.K. Maloney, S. Huang *et al.*, 2009 Copy number changes of the microcephalin 1 gene (MCPH1) in patients with autism spectrum disorders. *Clinical Genetics* 76 (4):348-356.

Peca, J., C. Feliciano, J.T. Ting, W. Wang, M.F. Wells *et al.*, 2011 Shank3 mutant mice display autistic-like behaviours and striatal dysfunction. *Nature* 472 (7344):437-442.

Pinto, D., A.T. Pagnamenta, L. Klei, R. Anney, D. Merico *et al.*, 2010 Functional impact of global rare copy number variation in autism spectrum disorders. *Nature* 466 (7304):368-372.

Rosenberg, M.J., R. Agarwala, G. Bouffard, J. Davis, G. Fiermonte *et al.*, 2002 Mutant deoxynucleotide carrier is associated with congenital microcephaly. *Nature Genetics* 32 (1):175-179.

Sadakata, T., M. Washida, Y. Iwayama, S. Shoji, Y. Sato *et al.*, 2007 Autistic-like phenotypes in Cadps2-knockout mice and aberrant CADPS2 splicing in autistic patients. *Journal of Clinical Investigation* 117 (4):931-943.

Savelieva, K.V., I. Rajan, K.B. Baker, P. Vogel, W. Jarman *et al.*, 2008 Learning and memory impairment in Eph receptor A6 knockout mice. *Neuroscience Letters* 438 (2):205-209.

Spiegel, R., A. Shaag, S. Edvardson, H. Mandel, P. Stepensky *et al.*, 2009 SLC25A19 mutation as a cause of neuropathy and bilateral striatal necrosis. *Annals of Neurology* 66 (3):419-424.

Thompson, B.A., V. Tremblay, G. Lin, and D.A. Bochar, 2008 CHD8 is an ATP-dependent chromatin remodeling factor that regulates beta-catenin target genes. *Molecular and Cellular Biology* 28 (12):3894-3904.

Trimborn, M., S.M. Bell, C. Felix, Y. Rashid, H. Jafri *et al.*, 2004 Mutations in microcephalin cause aberrant regulation of chromosome condensation. *American Journal of Human Genetics* 75 (2):261-266.

Tucci, V., T. Kleefstra, A. Hardy, I. Heise, S. Maggi *et al.*, 2014 Dominant beta-catenin mutations cause intellectual disability with recognizable syndromic features. *Journal of Clinical Investigation* 124 (4):1468-1482.

Waite, K.A., and C. Eng, 2002 Protean PTEN: form and function. *American Journal of Human Genetics* 70 (4):829-844.

Wang, X., P.A. McCoy, R.M. Rodriguiz, Y. Pan, H.S. Je *et al.*, 2011 Synaptic dysfunction and abnormal behaviors in mice lacking major isoforms of Shank3. *Human Molecular Genetics* 20 (15):3093-3108.

Weiss, L.A., A. Escayg, J.A. Kearney, M. Trudeau, B.T. MacDonald *et al.*, 2003 Sodium channels SCN1A, SCN2A and SCN3A in familial autism. *Molecular Psychiatry* 8 (2):186-194.

Willert, K., and R. Nusse, 1998 Beta-catenin: a key mediator of Wnt signaling. *Current Opinion in Genetics and Development* 8 (1):95-102.

Wu, Y., F.V. Bolduc, K. Bell, T. Tully, Y. Fang *et al.*, 2008 A Drosophila model for Angelman syndrome. *Proceedings of the National Academy of Sciences of the United States of America* 105 (34):12399-12404.

Xu, X., J. Lee, and D.F. Stern, 2004 Microcephalin is a DNA damage response protein involved in regulation of CHK1 and BRCA1. *Journal of Biological Chemistry* 279 (33):34091-34094.
