# Supplementary material for: Quantitative Assessment of Eye Phenotypes for Functional Genetic Studies Using Drosophila melanogaster
Source: G3 (Bethesda). 2016 Mar 18;6(5):1427–37. doi: 10.1534/g3.116.027060 (PMC4856093; doi:10.1534/g3.116.027060)
Supplement: Supplemental Material [file supp_g3.116.027060_TableS1.pdf]

Table S1. Stock list of candidate genotypes prioritized from the deficiency screen of modifiers of UAS-*so*

Stock #	Genotype
BL442	Df(2R)CX1, wg12 b1 pr1/SM1
BL727	Df(1)g, f[1] B[1]/In(1)AM
BL1931	Df(3R)by10, red1 e1/TM3, Sb1 Ser1
BL2366	Df(3R)XTA1, Diap11 st1 kniri-1 rnroe-1 p1/Dp(3;3)M95A+13, st1 e1
BL2414	Df(2L)spdj2, wgspd-j2/CyO, P{ftz/lacB}E3
BL3520	wa Nfa-g; Df(2R)Jp8, w+/CyO
BL3347	Df(1)sd72b/FM7c
BL7144	Df(2L)BSC37, dpp[EP2232]/CyO
BL7659	w1118; Df(3R)Exel6180, P{XP-U}Exel6180/TM6B, Tb1
BL7689	w1118; Df(3R)Exel6211, P{XP-U}Exel6211/TM6B, Tb1
BL8674	w1118; Df(2L)BSC109/CyO
BL8925	w1118; Df(3R)ED6316, P{3'.RS5+3.3'}ED6316/TM6C, cu1 Sb1
BL18322	w1118; PBac{WH}Cpsf100f00376/TM6B, Tb1
BL25005	w1118; Df(3R)BSC501/TM6C, Sb1 cu1
BL27378	w1118; Df(3R)BSC806, P+PBac{XP3.RB5}BSC806/TM6C, Sb1 cu1
BL34665	y1 sc* v1; P{TRiP.HMS01142}attP2
