# Supplementary material for: Quantitative Assessment of Eye Phenotypes for Functional Genetic Studies Using Drosophila melanogaster
Source: G3 (Bethesda). 2016 Mar 18;6(5):1427–37. doi: 10.1534/g3.116.027060 (PMC4856093; doi:10.1534/g3.116.027060)
Supplement: Supplemental Material [file supp_g3.116.027060_TableS2.pdf]

Table S2. Primers used for quantitative real time PCR

**Primers**	**Sequence**
para (forward)	5' AGCTATACCGTCCATCTTCAATG 3'
para (reverse)	5' TTTGGTATGATCTCGTGGCTG 3'
prosap (forward)	5' CCGGAGCTGAATGTCTACAAG 3'
prosap (reverse)	5' GCAAACAGGCCATAGTTGAAG 3'
kismet (forward)	5' AAGACGTTCATCTGGGACTTG 3'
kismet (reverse)	5' GTTTGACTTTCCACCGTTGC 3'
caps (forward)	5' TCCCACAAACCAACTCCAC 3'
caps (reverse)	5' CAAAAGTACAAGGATCGGTGC 3'
dube3a (forward)	5' GATTGAAATGCTGGTCTGCG 3'
dube3a (reverse)	5' CACTATGCTCCAGAAGTCCTG 3'
arm (forward)	5' CAGAATCGAACCATGTCGCAT 3'
arm (reverse)	5' CGGCAGATCAGGTGGATTGT 3'
dpten (forward)	5' TCCAATGTTGTAGCCGTGGA 3'
dpten (reverse)	5' GGTACCGGTTCTGCCCTTTC 3'
