# Supplementary material for: Quantitative Assessment of Eye Phenotypes for Functional Genetic Studies Using Drosophila melanogaster
Source: G3 (Bethesda). 2016 Mar 18;6(5):1427–37. doi: 10.1534/g3.116.027060 (PMC4856093; doi:10.1534/g3.116.027060)
Supplement: Supplemental Material [file supp_g3.116.027060_FigureS1.pdf]

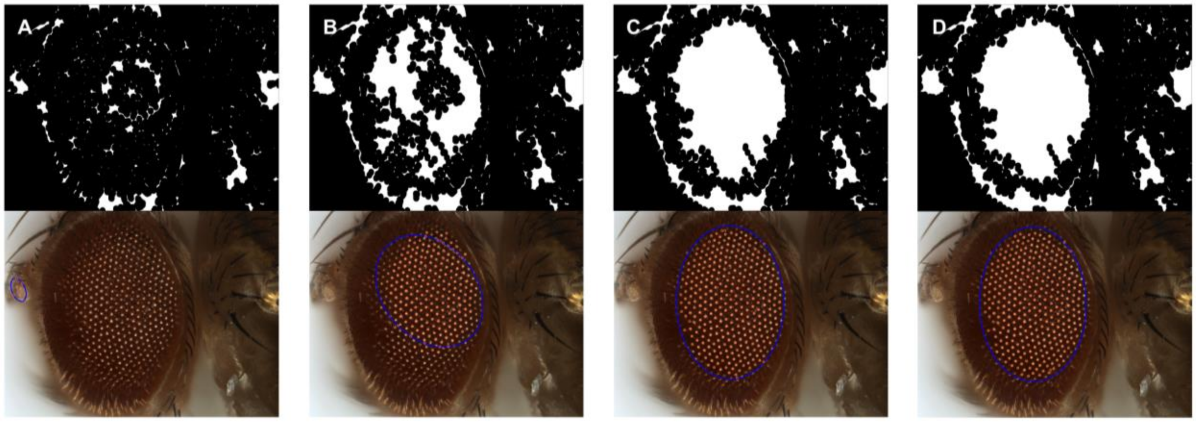

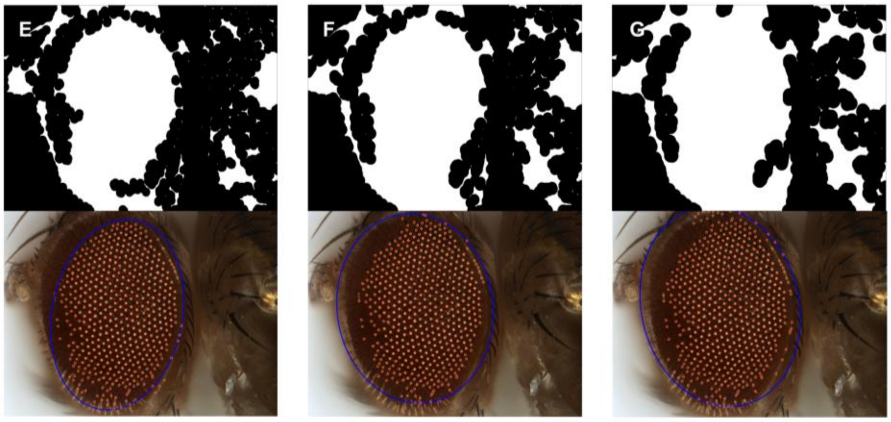

	Size of SE for Top Hat operation	Size of SE for Closing operation
a	(7,7)	(50,60)
b	(11,11)	(50,60)
c	(15,15)	(50,60)
d	(19,19)	(50,60)
e	(15,15)	(70,80)
f	(15,15)	(90,100)
g	(15,15)	(110,120)

Figure S1. Optimization of eye area identification.
Different combinations of structuring elements (SE) sizes were tested for the Top Hat and Closing transformation operations to optimize the accuracy of eye area localization (**A-G**). All sizes of structuring elements used for the transformation operations are shown in the table.
