# Supplementary material for: Quantitative Assessment of Eye Phenotypes for Functional Genetic Studies Using Drosophila melanogaster
Source: G3 (Bethesda). 2016 Mar 18;6(5):1427–37. doi: 10.1534/g3.116.027060 (PMC4856093; doi:10.1534/g3.116.027060)
Supplement: Supplemental Material [file supp_g3.116.027060_TableS3.pdf]

Table S3. A list of neurodevelopmental genes assessed for eye phenotypes

Human gene (Fly ortholog)	Function	Model organism studies	Phenotypes
SHANK3 (prosap): SH3 and multiple ankyrin repeat domains 3	*SHANK3* is a synaptic scaffolding protein enriched in the postsynaptic density (PSD) of excitatory synapses (Kreienkamp 2008). It plays important roles in synaptogenesis, synaptic plasticity and regulation of dendritic spine morphology (Boeckers et al. 2002).	Mice with *Shank3* haploinsufficiency display defects in synaptic function and plasticity and reduced social interactions (Bozdagi et al. 2010). *Shank3* homozygous mutant mice exhibit abnormal social behavior, repetitive behavior, defects in learning and memory, morphological alterations in dendritic spines, and impaired long term potentiation (Peca et al. 2011; Wang et al. 2011)	ASD (Moessner et al. 2007; Gauthier et al. 2009), language and/or social communication impairment (Durand et al. 2007).
UBE3A (dube3a): Ubiquitin protein ligase E3A	UBE3A is part of the ubiquitin protein degradation system, which accepts ubiquitin from an E2 ubiquitin-conjugating enzyme in the form of a thioester and transfers it to its substrates (Hershko and Ciechanover 1998)	*Ube3A* maternal-deficient mice display reduced brain weight, ataxia, motor defects and behavioral defects (Jiang et al. 1998; Heck et al. 2008). Lack of *dube3A* in *Drosophila* leads to motor defects, abnormal circadian rhythms, long-term memory defects and abnormalities in dendritic arborization in the peripheral nervous system (Lu et al. 2009; Wu et al. 2008).	ASD, intellectual disability and developmental delay (Nurmi et al. 2001; Kelleher et al. 2012; Glessner et al. 2009).
SCN1A (para): Sodium Channel, Voltage-Gated, Type I, Alpha Subunit	*SCN1A* encodes a voltage gated sodium channel that is essential for generation and propagation of action potential (Catterall et al. 2010)	Mice with a loss-of-function nonsense mutation in *Scn1a* develop epileptic seizures within the first postnatal month (Ogiwara et al. 2007). Mice with *Scn1a* haploinsufficiency exhibit hyperactivity, stereotyped behaviors, social interaction deficits and impaired context-dependent	Dravet's syndrome (childhood neuropsychiatric disorder including intractable seizures, cognitive deficits and ASD) (Claes et al. 2001;

		spatial memory and these features are rescued by a low-dose clonazepam, a positive allosteric modulator of GABA(A) receptors (Han et al. 2012).	Frosk et al. 2013), epilepsy (Epi et al. 2013; Kasperaviciute et al. 2013) and ASD (Weiss et al. 2003; O'Roak et al. 2011).
PTEN (dpten): Phosphatase and Tensin Homolog	*PTEN* is a tumor suppressor with lipid phosphatase activity (PMID: 10564676).It is a protein phosphatase, that can dephosphorylate both serine and threonine residues. It plays an important role in PI3 kinase/AKT pathway, MAPK pathway and in the mediation of growth arrest (Ali et al. 1999).	*Pten* haploinsufficient mice exhibit social defects and repetitive behavior (PMID: 22900024). Mice with CNS deletion of Pten displayed abnormal social behavior and defects in social interactions (Waite and Eng 2002)	Cancer (Napoli et al. 2012; Kwon et al. 2006), Macrocephaly, autism (Waite and Eng 2002; Goffin et al. 2001; O'Roak et al. 2012a), learning disabilities (Busa et al. 2013) and epilepsy (Epi et al. 2013).
CADPS2 (caps): Calcium dependent activator protein for secretion	*CADPS2* regulates exocytosis of dense core vesicles (Grishanin et al. 2004)	*Cadps2* knockout mice display impaired cerebellar development and function and autistic-like cellular and behavioral phenotypes (Sadakata et al. 2007)	Autism (Cisternas et al. 2003; Okamoto et al. 2011; Girirajan et al. 2013), Intellectual disability (Bonora et al. 2014).
NRXN1 (nrxn1): Neurexin 1	*Neurexin 1* is a synaptic cell adhesion protein, involved in synaptic formation and function (Chen et al. 2010)	*Nrxn1* deficient mice exhibit defect in excitatory synaptic strength, decrease in prepulse inhibition, an increase in grooming behaviors, an impairment in nest-building activity, and an improvement in motor learning (Etherton et al. 2009).	Autism, intellectual disability, speech delays, seizures, poor muscle tone, unusual facial features (Dabell et al. 2013; Bena et al. 2013).
MCPH1 (mcph1):	*MCPH1* is regulator of chromosome	*Mcph1* null mouse model demonstrates its	Autism (Ozgen et al.

Microcephalin 1	condensation (Trimborn et al. 2004) and is involved in DNA damage induced cellular responses (Xu et al. 2004). May play a role in neurogenesis and regulation of the size of the cerebral cortex (Mahmood et al. 2011).	role in maintaining genomic stability and in regulating programmed and IR-induced DNA damage response (Liang et al. 2010).	2009; Neale et al. 2012), primary microcephaly.
LGR5 (rk): Leucine-rich repeat containing G protein-coupled receptor 5	*LGR5* functions as receptors for R-spondins and regulates canonical Wnt/beta-catenin signaling (Carmon et al. 2011). LGR5 has also been validated as a stem cell marker of the intestinal epithelium (Barker et al. 2007) and the hair follicle (Jaks et al. 2008).	Knockout of *Lgr5* in the mouse leads to total neonatal lethality characterized by gastrointestinal tract dilation and accompanied by ankyloglossia (Morita et al. 2004).	Deletion of human chromosomal region 12q21.1 containing LGR5 is associated with ASD (Thompson et al. 2008).
CHD8 (kismet): Chromodomain-Helicase-DNA-Binding Protein 8	*CHD8* is a DNA helicase that acts as a chromatin-remodeling factor and regulates transcription. Acts as a negative regulator of Wnt signaling pathway by regulating beta-catenin activity (Thompson et al. 2008).	*Chd8* null mice die during early embryogenesis due to widespread apoptosis. CHD8 plays a role in regulating tumor suppressor p53 activity and prevents apoptosis mediated by p53 (Nishiyama et al. 2009).	Autism, gastrointestinal disorders, macrocephaly and characteristic facial features, including wide-set eyes, large ears, broad foreheads and noses (Bernier et al. 2014).
CTNNB1 (arm): Catenin (cadherin-associated protein), beta 1	*CTNNB1* is a pivotal component of the canonical Wnt signaling pathway. Wnt signal stabilizes the beta-catenin, which then accumulates in the cytoplasm and is subsequently translocated to the nucleus, where it	Complete knockout of *Ctnnb1* is embryonic lethal (Haegel et al. 1995). Mutant *Cnnb1* mice with deletion restricted to the dorsal telencephalon, surivive to adulthood and show increased susceptibility to seizures and cortical malformation (Campos et al.	Autism, intellectual disability, low muscle tone and microcephaly (Dubruc et al. 2014; Tucci et al. 2014; de Ligt et al. 2012; O'Roak et al.

	interacts with members of the TCF family of transcription factors and induces the transcription of Wnt target genes (Willert and Nusse 1998).	2004)	2012b)
EPHA6 (eph): Ephrin Type-A Receptor 6	*EPHA6* is a member of Eph family of tyrosine kinases that are involved in development of neuronal projection pathways (Martinez and Soriano 2005)	*EphA6* knockout mice exhibit learning and memory impairments (Savelieva et al. 2008)	ASD (Girirajan et al. 2013; Pinto et al. 2010).
SLC25A19 (tpc1): solute carrier family 25 (mitochondrial thiamine pyrophosphate carrier), member 19	*SLC25A19* is involved in the transport of thiamine pyrophosphate (Kang and Samuels 2008)	*Slc25a19* knockout mice display mitochondrial pyrophosphate depletion, embryonic lethality, CNS malformations and anemia (Lindhurst et al. 2006).	Amish lethal microcephaly (Rosenberg et al. 2002), bilateral striatal necrosis and chronic progressive polyneuropathy (Spiegel et al. 2009).
