# Supplementary material for: Quantitative Assessment of Eye Phenotypes for Functional Genetic Studies Using Drosophila melanogaster
Source: G3 (Bethesda). 2016 Mar 18;6(5):1427–37. doi: 10.1534/g3.116.027060 (PMC4856093; doi:10.1534/g3.116.027060)
Supplement: Supplemental Material [file supp_g3.116.027060_TableS4.pdf]

Table S4. *Drosophila* orthologs of human neurodevelopmental genes and the qualitative rank order of their eye phenotypes

Number	Human Gene	*Drosophila* ortholog	Stock#	Rank order
1	*SHANK3*	*prosap*	21218	1
2	*LGR5*	*rk*	105360	1
3	*SCN1A*	*para*	6132	2
4	*UBE3A*	*dube3a*	45876	2
5	*EPHA6*	*eph*	6545	2
6	*LGR5*	*rk*	29932	2
7	*SCN1A*	*para*	104775	3
8	*SLC25A19*	*tpc1*	6005	3
9	*SCN1A*	*para*	6131	3.5
10	*LGR5*	*rk*	29931	4
11	*MCPH1*	*mcph1*	28100	4
12	*PTEN*	*dpten*	101475	4
13	*CADPS2*	*caps*	25292	5
14	*CHD8*	*kismet*	46685	6
15	*SHANK3*	*prosap*	103592	6.5
16	*NRXN-1*	*nrx-1*	4306	7
17	*MCPH1*	*mcph1*	106261	8
18	*CTNNB1*	*arm*	107344	8
19	*CADPS2*	*caps*	25291	8.5
20	*UBE3A*	*dube3a*	100130	9
21	*PTEN*	*dpten*	35731	9
