# Supplementary material for: Quantitative Assessment of Eye Phenotypes for Functional Genetic Studies Using Drosophila melanogaster
Source: G3 (Bethesda). 2016 Mar 18;6(5):1427–37. doi: 10.1534/g3.116.027060 (PMC4856093; doi:10.1534/g3.116.027060)
Supplement: Supplemental Material [file supp_g3.116.027060_TableS5.pdf]

Table S5. Student *t* test comparing eye phenotypes of neurodevelopmental genes with controls

Genotypes	Number of samples	Two-tailed p value	Two-tailed p value (After multiple testing correction)
Control	22	1	-
$prosap^{21218}$	18	0.498	-
rk^{105360}	22	3.93×10^{-04}	8.25×10^{-03}
$dube3a^{45876}$	25	4.06×10^{-06}	8.52×10^{-05}
eph^{6545}	22	1.97×10^{-06}	4.14×10^{-05}
rk^{29932}	12	3.28×10^{-06}	6.89×10^{-05}
$para^{6132}$	17	4.76×10^{-08}	9.99×10^{-07}
$para^{104775}$	21	2.49×10^{-08}	5.22×10^{-07}
$tpc1^{6005}$	24	3.67×10^{-11}	7.70×10^{-10}
$para^{6131}$	20	1.11×10^{-12}	2.34×10^{-11}
rk^{29931}	9	4.89×10^{-11}	1.03×10^{-09}
$mcph1^{28100}$	20	4.74×10^{-12}	9.95×10^{-11}
$dpten^{101475}$	18	6.21×10^{-15}	1.30×10^{-13}
$caps^{25292}$	15	3.37×10^{-13}	7.07×10^{-12}
$prosap^{103592}$	30	2.76×10^{-23}	5.79×10^{-22}
$nrx1^{4306}$	17	4.75×10^{-21}	9.98×10^{-20}
$kismet^{46685}$	27	2.17×10^{-21}	4.55×10^{-20}
$mcph1^{106261}$	21	1.46×10^{-24}	3.07×10^{-23}
arm^{107344}	25	2.22×10^{-21}	4.66×10^{-20}
$caps^{25291}$	17	3.82×10^{-22}	8.03×10^{-21}
$dpten^{35731}$	17	4.55×10^{-15}	9.55×10^{-14}
$dube3a^{100130}$	24	3.23×10^{-26}	6.79×10^{-25}

Multiple testing corrections were performed using Bonferroni method
