# Supplementary material for: Quantitative Assessment of Eye Phenotypes for Functional Genetic Studies Using Drosophila melanogaster
Source: G3 (Bethesda). 2016 Mar 18;6(5):1427–37. doi: 10.1534/g3.116.027060 (PMC4856093; doi:10.1534/g3.116.027060)
Supplement: Supplemental Material [file supp_g3.116.027060_TableS6.pdf]

Table S6. Student *t* test comparing phenotypic scores of eye phenotypes at 28°C to that at 30°C

Fly genotype	Number of samples (30°C)	Number of samples (28°C)	Student t test, two-tailed p value	Corrected two-tailed p value
Control	22	14	0.009	0.19
$prosap^{21218}$	18	17	3.81×10^{-07}	6.86×10^{-06}
$dube3a^{45876}$	25	29	7.71×10^{-19}	1.92×10^{-17}
$para^{6131}$	17	24	8.98×10^{-16}	1.52×10^{-14}
$para^{6132}$	20	23	4.25×10^{-19}	8.50×10^{-18}
$dpten^{101475}$	18	23	1.29×10^{-11}	2.33×10^{-10}
$caps^{25292}$	15	24	5.74×10^{-07}	8.61×10^{-06}
$prosap^{103592}$	30	26	3.84×10^{-28}	1.15×10^{-26}
arm^{107344}	27	24	1.51×10^{-19}	4.09×10^{-18}
$caps^{25291}$	25	17	3.77×10^{-05}	0.00094
$dpten^{35731}$	17	20	8.96×10^{-10}	1.52×10^{-08}
$kismet^{46685}$	17	20	3.60×10^{-12}	6.12×10^{-11}
$dube3a^{100130}$	24	25	6.64×10^{-26}	1.59×10^{-24}

Student t test was used to calculate two tailed p values. Multiple-testing correction was performed using Bonferroni method.
