# Supplementary material for: Quantitative Assessment of Eye Phenotypes for Functional Genetic Studies Using Drosophila melanogaster
Source: G3 (Bethesda). 2016 Mar 18;6(5):1427–37. doi: 10.1534/g3.116.027060 (PMC4856093; doi:10.1534/g3.116.027060)
Supplement: Supplemental Material [file supp_g3.116.027060_TableS7.pdf]

Table S7. Student *t* test comparing the phenotypic scores of modifiers of UAS-*so* with phenotypic scores from UAS-*so* alone

Genotype	Number of SEM images	One-tailed p value*	Corrected one-tailed p value[a]	Two-tailed p value*	Corrected two-tailed p value[a]	Modifier
eyG4_UAS-so_BL27378	2	0.1774	-	0.35	-	Strong enhancer
eyG4_UAS-so_BL7689	2	0.1078	-	0.21	-	Strong enhancer
eyG4_UAS-so_BL18322	2	0.0855	0.76	0.17	-	Moderate enhancer
eyG4_UAS-so_BL7659	2	0.0531	-	0.10	-	Mild enhancer
**eyG4_UAS-so**	**3**	**0.5000**	**-**	**-**	**-**	**-**
WT	10	-	-	-	-	-
eyG4_UAS-so_BL25005	2	0.2661	-	0.53	-	Mild suppressor
eyG4_UAS-so_BL2366	2	0.0103	0.09	0.02	0.18	Moderate suppressor
eyG4_UAS-so_BL8925	2	0.0086	0.07	0.0173	0.15	Moderate suppressor
eyG4_UAS-so_BL34665	2	0.0069	0.06	0.0137	0.12	Strong suppressor
GMR_UAS-so_BL442	2	0.0247	0.22	0.0495	0.44	Strong enhancer
GMR_UAS-so_BL2414	3	0.0027	0.023	0.0053	0.047	Strong enhancer
GMR_UAS-so_BL1931	2	0.0337	0.3	0.0674	-	Strong enhancer
GMR_UAS-so_BL3520	3	0.3100	-	0.62	-	Mild enhancer
**GMR_UAS-so**	**3**	**0.5000**	**-**		**-**	
GMR_UAS-so_BL7144	3	0.0003	0.0027	0.0006	0.005	Strong suppressor

GMR_UAS-so_BL8674	3	0.0003	0.0026	0.0006	0.005	Strong suppressor
GMR_UAS-so_BL)727	3	0.0002	0.0014	0.0003	0.0029	Strong suppressor
GMR_UAS-so_BL3347	2	0.0040	0.036	0.0081	0.072	Strong suppressor
WT	10	-	-	-	-	-

*Student *t* test was used for calculations. [a]Multiple testing corrections using Bonferroni method were applied.
