# Supplementary material for: Quantitative Assessment of Eye Phenotypes for Functional Genetic Studies Using Drosophila melanogaster
Source: G3 (Bethesda). 2016 Mar 18;6(5):1427–37. doi: 10.1534/g3.116.027060 (PMC4856093; doi:10.1534/g3.116.027060)
Supplement: Supplemental Material [file supp_g3.116.027060_TableS8.pdf]

Table S8. Features and limitations of Flynotyper

**Phenotypes identifiable**	**Flynotyper**
Glossy eye	Yes
Rough eye	Yes
Crinkled eye	Yes
Necrotic eye	Yes
Bristle integrity	No
Ommatidial size	No
Loss of pigmentation	No
Size of the eye	No
**Technical features**	**Flynotyper**
Quantification of eye roughness	Yes
Automatic identification of region of interest	Yes
Requires a manual step prior to processing the images	No
Robust performance with different image acquisition set ups	Yes
