# Supplementary material for: Quantitative Assessment of Eye Phenotypes for Functional Genetic Studies Using Drosophila melanogaster
Source: G3 (Bethesda). 2016 Mar 18;6(5):1427–37. doi: 10.1534/g3.116.027060 (PMC4856093; doi:10.1534/g3.116.027060)
Supplement: Supplemental Material [file supp_g3.116.027060_SupplementalReferences.pdf]

References

Ali, I.U., L.M. Schriml, and M. Dean, 1999 Mutational spectra of PTEN/MMAC1 gene: a tumor suppressor with lipid phosphatase activity. *Journal of the National Cancer Institute* 91 (22):1922-1932.

Ambegaokar, S.S., and G.R. Jackson, 2011 Functional genomic screen and network analysis reveal novel modifiers of tauopathy dissociated from tau phosphorylation. *Human Molecular Genetics* 20 (24):4947-4977.

Barker, N., J.H. van Es, J. Kuipers, P. Kujala, M. van den Born *et al.*, 2007 Identification of stem cells in small intestine and colon by marker gene Lgr5. *Nature* 449 (7165):1003-1007.

Bena, F., D.L. Bruno, M. Eriksson, C. van Ravenswaaij-Arts, Z. Stark *et al.*, 2013 Molecular and clinical characterization of 25 individuals with exonic deletions of NRXN1 and comprehensive review of the literature. *American Journal of Medical Genetics. Part B, Neuropsychiatric Genetics* 162B (4):388-403.

Bernier, R., C. Golzio, B. Xiong, H.A. Stessman, B.P. Coe *et al.*, 2014 Disruptive CHD8 mutations define a subtype of autism early in development. *Cell* 158 (2):263-276.

Boeckers, T.M., J. Bockmann, M.R. Kreutz, and E.D. Gundelfinger, 2002 ProSAP/Shank proteins - a family of higher order organizing molecules of the postsynaptic density with an emerging role in human neurological disease. *Journal of Neurochemistry* 81 (5):903-910.

Bonora, E., C. Graziano, F. Minopoli, E. Bacchelli, P. Magini *et al.*, 2014 Maternally inherited genetic variants of CADPS2 are present in autism spectrum disorders and intellectual disability patients. *EMBO Mol Med* 6 (6):795-809.

Bozdagi, O., T. Sakurai, D. Papapetrou, X. Wang, D.L. Dickstein *et al.*, 2010 Haploinsufficiency of the autism-associated Shank3 gene leads to deficits in synaptic function, social interaction, and social communication. *Mol Autism* 1 (1):15.

Busa, T., B. Chabrol, O. Perret, M. Longy, and N. Philip, 2013 Novel PTEN germline mutation in a family with mild phenotype: difficulties in genetic counseling. *Gene* 512 (2):194-197.

Campos, V.E., M. Du, and Y. Li, 2004 Increased seizure susceptibility and cortical malformation in beta-catenin mutant mice. *Biochemical and Biophysical Research Communications* 320 (2):606-614.

Carmon, K.S., X. Gong, Q. Lin, A. Thomas, and Q. Liu, 2011 R-spondins function as ligands of the orphan receptors LGR4 and LGR5 to regulate Wnt/beta-catenin signaling. *Proceedings of the National Academy of Sciences of the United States of America* 108 (28):11452-11457.

Catterall, W.A., F. Kalume, and J.C. Oakley, 2010 NaV1.1 channels and epilepsy. *J Physiol* 588 (Pt 11):1849-1859.

Chen, K., E.O. Gracheva, S.C. Yu, Q. Sheng, J. Richmond *et al.*, 2010 Neurexin in embryonic Drosophila neuromuscular junctions. *PLoS ONE* 5 (6):e11115.

Cisternas, F.A., J.B. Vincent, S.W. Scherer, and P.N. Ray, 2003 Cloning and characterization of human CADPS and CADPS2, new members of the Ca2+-dependent activator for secretion protein family. *Genomics* 81 (3):279-291.

Claes, L., J. Del-Favero, B. Ceulemans, L. Lagae, C. Van Broeckhoven *et al.*, 2001 De novo mutations in the sodium-channel gene SCN1A cause severe myoclonic epilepsy of infancy. *American Journal of Human Genetics* 68 (6):1327-1332.

Dabell, M.P., J.A. Rosenfeld, P. Bader, L.F. Escobar, D. El-Khechen *et al.*, 2013 Investigation of NRXN1 deletions: clinical and molecular characterization. *American Journal of Medical Genetics. Part A* 161A (4):717-731.

de Ligt, J., M.H. Willemsen, B.W. van Bon, T. Kleefstra, H.G. Yntema *et al.*, 2012 Diagnostic Exome Sequencing in Persons with Severe Intellectual Disability. *New England Journal of Medicine* 367 (20):1921-1929.

Dubruc, E., A. Putoux, A. Labalme, C. Rougeot, D. Sanlaville *et al.*, 2014 A new intellectual disability syndrome caused by CTNNB1 haploinsufficiency. *American Journal of Medical Genetics. Part A* 164A (6):1571-1575.

Durand, C.M., C. Betancur, T.M. Boeckers, J. Bockmann, P. Chaste *et al.*, 2007 Mutations in the gene encoding the synaptic scaffolding protein SHANK3 are associated with autism spectrum disorders. *Nature Genetics* 39 (1):25-27.

Epi, K.C., P. Epilepsy Phenome/Genome, A.S. Allen, S.F. Berkovic, P. Cossette *et al.*, 2013 De novo mutations in epileptic encephalopathies. *Nature* 501 (7466):217-221.

Etherton, M.R., C.A. Blaiss, C.M. Powell, and T.C. Sudhof, 2009 Mouse neurexin-1alpha deletion causes correlated electrophysiological and behavioral changes consistent with cognitive impairments. *Proceedings of the National Academy of Sciences of the United States of America* 106 (42):17998-18003.

Frosk, P., A.A. Mhanni, and M.F. Rafay, 2013 SCN1A mutation associated with intractable myoclonic epilepsy and migraine headache. *Journal of Child Neurology* 28 (3):389-391.

Gauthier, J., D. Spiegelman, A. Piton, R.G. Lafreniere, S. Laurent *et al.*, 2009 Novel de novo SHANK3 mutation in autistic patients. *American Journal of Medical Genetics. Part B, Neuropsychiatric Genetics* 150B (3):421-424.

Girirajan, S., M.Y. Dennis, C. Baker, M. Malig, B.P. Coe *et al.*, 2013 Refinement and discovery of new hotspots of copy-number variation associated with autism spectrum disorder. *American Journal of Human Genetics* 92 (2):221-237.

Glessner, J.T., K. Wang, G. Cai, O. Korvatska, C.E. Kim *et al.*, 2009 Autism genome-wide copy number variation reveals ubiquitin and neuronal genes. *Nature* 459 (7246):569-573.

Goffin, A., L.H. Hoefsloot, E. Bosgoed, A. Swillen, and J.P. Fryns, 2001 PTEN mutation in a family with Cowden syndrome and autism. *American Journal of Medical Genetics* 105 (6):521-524.

Grishanin, R.N., J.A. Kowalchyk, V.A. Klenchin, K. Ann, C.A. Earles *et al.*, 2004 CAPS acts at a prefusion step in dense-core vesicle exocytosis as a PIP2 binding protein. *Neuron* 43 (4):551-562.

Haegel, H., L. Larue, M. Ohsugi, L. Fedorov, K. Herrenknecht *et al.*, 1995 Lack of beta-catenin affects mouse development at gastrulation. *Development* 121 (11):3529-3537.

Han, S., C. Tai, R.E. Westenbroek, F.H. Yu, C.S. Cheah *et al.*, 2012 Autistic-like behaviour in Scn1a+/- mice and rescue by enhanced GABA-mediated neurotransmission. *Nature* 489 (7416):385-390.

Heck, D.H., Y. Zhao, S. Roy, M.S. LeDoux, and L.T. Reiter, 2008 Analysis of cerebellar function in Ube3a-deficient mice reveals novel genotype-specific behaviors. *Human Molecular Genetics* 17 (14):2181-2189.

Hershko, A., and A. Ciechanover, 1998 The ubiquitin system. *Annual Review of Biochemistry* 67:425-479.

Jaks, V., N. Barker, M. Kasper, J.H. van Es, H.J. Snippert *et al.*, 2008 Lgr5 marks cycling, yet long-lived, hair follicle stem cells. *Nature Genetics* 40 (11):1291-1299.

Jiang, Y.H., D. Armstrong, U. Albrecht, C.M. Atkins, J.L. Noebels *et al.*, 1998 Mutation of the Angelman ubiquitin ligase in mice causes increased cytoplasmic p53 and deficits of contextual learning and long-term potentiation. *Neuron* 21 (4):799-811.

Kang, J., and D.C. Samuels, 2008 The evidence that the DNC (SLC25A19) is not the mitochondrial deoxyribonucleotide carrier. *Mitochondrion* 8 (2):103-108.

Kasperaviciute, D., C.B. Catarino, M. Matarin, C. Leu, J. Novy *et al.*, 2013 Epilepsy, hippocampal sclerosis and febrile seizures linked by common genetic variation around SCN1A. *Brain* 136 (Pt 10):3140-3150.

Kelleher, R.J., 3rd, U. Geigenmuller, H. Hovhannisyan, E. Trautman, R. Pinard *et al.*, 2012 High-throughput sequencing of mGluR signaling pathway genes reveals enrichment of rare variants in autism. *PLoS ONE* 7 (4):e35003.

Kreienkamp, H.J., 2008 Scaffolding proteins at the postsynaptic density: shank as the architectural framework. *Handb Exp Pharmacol* (186):365-380.

Kwon, C.H., B.W. Luikart, C.M. Powell, J. Zhou, S.A. Matheny *et al.*, 2006 Pten regulates neuronal arborization and social interaction in mice. *Neuron* 50 (3):377-388.

Liang, Y., H. Gao, S.Y. Lin, G. Peng, X. Huang *et al.*, 2010 BRIT1/MCPH1 is essential for mitotic and meiotic recombination DNA repair and maintaining genomic stability in mice. *PLoS Genetics* 6 (1):e1000826.

Lindhurst, M.J., G. Fiermonte, S. Song, E. Struys, F. De Leonardis *et al.*, 2006 Knockout of Slc25a19 causes mitochondrial thiamine pyrophosphate depletion, embryonic lethality, CNS malformations, and anemia. *Proceedings of the National Academy of Sciences of the United States of America* 103 (43):15927-15932.

Lu, Y., F. Wang, Y. Li, J. Ferris, J.A. Lee *et al.*, 2009 The Drosophila homologue of the Angelman syndrome ubiquitin ligase regulates the formation of terminal dendritic branches. *Human Molecular Genetics* 18 (3):454-462.

Mahmood, S., W. Ahmad, and M.J. Hassan, 2011 Autosomal Recessive Primary Microcephaly (MCPH): clinical manifestations, genetic heterogeneity and mutation continuum. *Orphanet Journal of Rare Diseases* 6:39.

Martinez, A., and E. Soriano, 2005 Functions of ephrin/Eph interactions in the development of the nervous system: emphasis on the hippocampal system. *Brain Research. Brain Research Reviews* 49 (2):211-226.

Moessner, R., C.R. Marshall, J.S. Sutcliffe, J. Skaug, D. Pinto *et al.*, 2007 Contribution of SHANK3 mutations to autism spectrum disorder. *American Journal of Human Genetics* 81 (6):1289-1297.

Morita, H., S. Mazerbourg, D.M. Bouley, C.W. Luo, K. Kawamura *et al.*, 2004 Neonatal lethality of LGR5 null mice is associated with ankyloglossia and gastrointestinal distension. *Molecular and Cellular Biology* 24 (22):9736-9743.

Napoli, E., C. Ross-Inta, S. Wong, C. Hung, Y. Fujisawa *et al.*, 2012 Mitochondrial dysfunction in Pten haplo-insufficient mice with social deficits and repetitive behavior: interplay between Pten and p53. *PLoS ONE* 7 (8):e42504.

Neale, B.M., Y. Kou, L. Liu, A. Ma'ayan, K.E. Samocha *et al.*, 2012 Patterns and rates of exonic de novo mutations in autism spectrum disorders. *Nature* 485 (7397):242-245.

Nishiyama, M., K. Oshikawa, Y. Tsukada, T. Nakagawa, S. Iemura *et al.*, 2009 CHD8 suppresses p53-mediated apoptosis through histone H1 recruitment during early embryogenesis. *Nature Cell Biology* 11 (2):172-182.

Nurmi, E.L., Y. Bradford, Y. Chen, J. Hall, B. Arnone *et al.*, 2001 Linkage disequilibrium at the Angelman syndrome gene UBE3A in autism families. *Genomics* 77 (1-2):105-113.

O'Roak, B.J., P. Deriziotis, C. Lee, L. Vives, J.J. Schwartz *et al.*, 2011 Exome sequencing in sporadic autism spectrum disorders identifies severe de novo mutations. *Nature Genetics* 43 (6):585-589.

O'Roak, B.J., L. Vives, W. Fu, J.D. Egertson, I.B. Stanaway *et al.*, 2012a Multiplex targeted sequencing identifies recurrently mutated genes in autism spectrum disorders. *Science* 338 (6114):1619-1622.

O'Roak, B.J., L. Vives, S. Girirajan, E. Karakoc, N. Krumm *et al.*, 2012b Sporadic autism exomes reveal a highly interconnected protein network of de novo mutations. *Nature* 485 (7397):246-250.

Ogiwara, I., H. Miyamoto, N. Morita, N. Atapour, E. Mazaki *et al.*, 2007 Nav1.1 localizes to axons of parvalbumin-positive inhibitory interneurons: a circuit basis for epileptic seizures in mice carrying an Scn1a gene mutation. *Journal of Neuroscience* 27 (22):5903-5914.

Okamoto, N., Y. Hatsukawa, K. Shimojima, and T. Yamamoto, 2011 Submicroscopic deletion in 7q31 encompassing CADPS2 and TSPAN12 in a child with autism spectrum disorder and PHPV. *American Journal of Medical Genetics. Part A* 155A (7):1568-1573.

Ozgen, H.M., E. van Daalen, P.F. Bolton, V.K. Maloney, S. Huang *et al.*, 2009 Copy number changes of the microcephalin 1 gene (MCPH1) in patients with autism spectrum disorders. *Clinical Genetics* 76 (4):348-356.

Peca, J., C. Feliciano, J.T. Ting, W. Wang, M.F. Wells *et al.*, 2011 Shank3 mutant mice display autistic-like behaviours and striatal dysfunction. *Nature* 472 (7344):437-442.

Pinto, D., A.T. Pagnamenta, L. Klei, R. Anney, D. Merico *et al.*, 2010 Functional impact of global rare copy number variation in autism spectrum disorders. *Nature* 466 (7304):368-372.

Rosenberg, M.J., R. Agarwala, G. Bouffard, J. Davis, G. Fiermonte *et al.*, 2002 Mutant deoxynucleotide carrier is associated with congenital microcephaly. *Nature Genetics* 32 (1):175-179.

Sadakata, T., M. Washida, Y. Iwayama, S. Shoji, Y. Sato *et al.*, 2007 Autistic-like phenotypes in Cadps2-knockout mice and aberrant CADPS2 splicing in autistic patients. *Journal of Clinical Investigation* 117 (4):931-943.

Savelieva, K.V., I. Rajan, K.B. Baker, P. Vogel, W. Jarman *et al.*, 2008 Learning and memory impairment in Eph receptor A6 knockout mice. *Neuroscience Letters* 438 (2):205-209.

Spiegel, R., A. Shaag, S. Edvardson, H. Mandel, P. Stepensky *et al.*, 2009 SLC25A19 mutation as a cause of neuropathy and bilateral striatal necrosis. *Annals of Neurology* 66 (3):419-424.

Thompson, B.A., V. Tremblay, G. Lin, and D.A. Bochar, 2008 CHD8 is an ATP-dependent chromatin remodeling factor that regulates beta-catenin target genes. *Molecular and Cellular Biology* 28 (12):3894-3904.

Trimborn, M., S.M. Bell, C. Felix, Y. Rashid, H. Jafri *et al.*, 2004 Mutations in microcephalin cause aberrant regulation of chromosome condensation. *American Journal of Human Genetics* 75 (2):261-266.

Tucci, V., T. Kleefstra, A. Hardy, I. Heise, S. Maggi *et al.*, 2014 Dominant beta-catenin mutations cause intellectual disability with recognizable syndromic features. *Journal of Clinical Investigation* 124 (4):1468-1482.

Waite, K.A., and C. Eng, 2002 Protean PTEN: form and function. *American Journal of Human Genetics* 70 (4):829-844.

Wang, X., P.A. McCoy, R.M. Rodriguiz, Y. Pan, H.S. Je *et al.*, 2011 Synaptic dysfunction and abnormal behaviors in mice lacking major isoforms of Shank3. *Human Molecular Genetics* 20 (15):3093-3108.

Weiss, L.A., A. Escayg, J.A. Kearney, M. Trudeau, B.T. MacDonald *et al.*, 2003 Sodium channels SCN1A, SCN2A and SCN3A in familial autism. *Molecular Psychiatry* 8 (2):186-194.

Willert, K., and R. Nusse, 1998 Beta-catenin: a key mediator of Wnt signaling. *Current Opinion in Genetics and Development* 8 (1):95-102.

Wu, Y., F.V. Bolduc, K. Bell, T. Tully, Y. Fang *et al.*, 2008 A Drosophila model for Angelman syndrome. *Proceedings of the National Academy of Sciences of the United States of America* 105 (34):12399-12404.

Xu, X., J. Lee, and D.F. Stern, 2004 Microcephalin is a DNA damage response protein involved in regulation of CHK1 and BRCA1. *Journal of Biological Chemistry* 279 (33):34091-34094.
