# Supplementary material for: Quantitative Assessment of Eye Phenotypes for Functional Genetic Studies Using Drosophila melanogaster
Source: G3 (Bethesda). 2016 Mar 18;6(5):1427–37. doi: 10.1534/g3.116.027060 (PMC4856093; doi:10.1534/g3.116.027060)
Supplement: Supplemental Material [file supp_g3.116.027060_FigureS9.pdf]

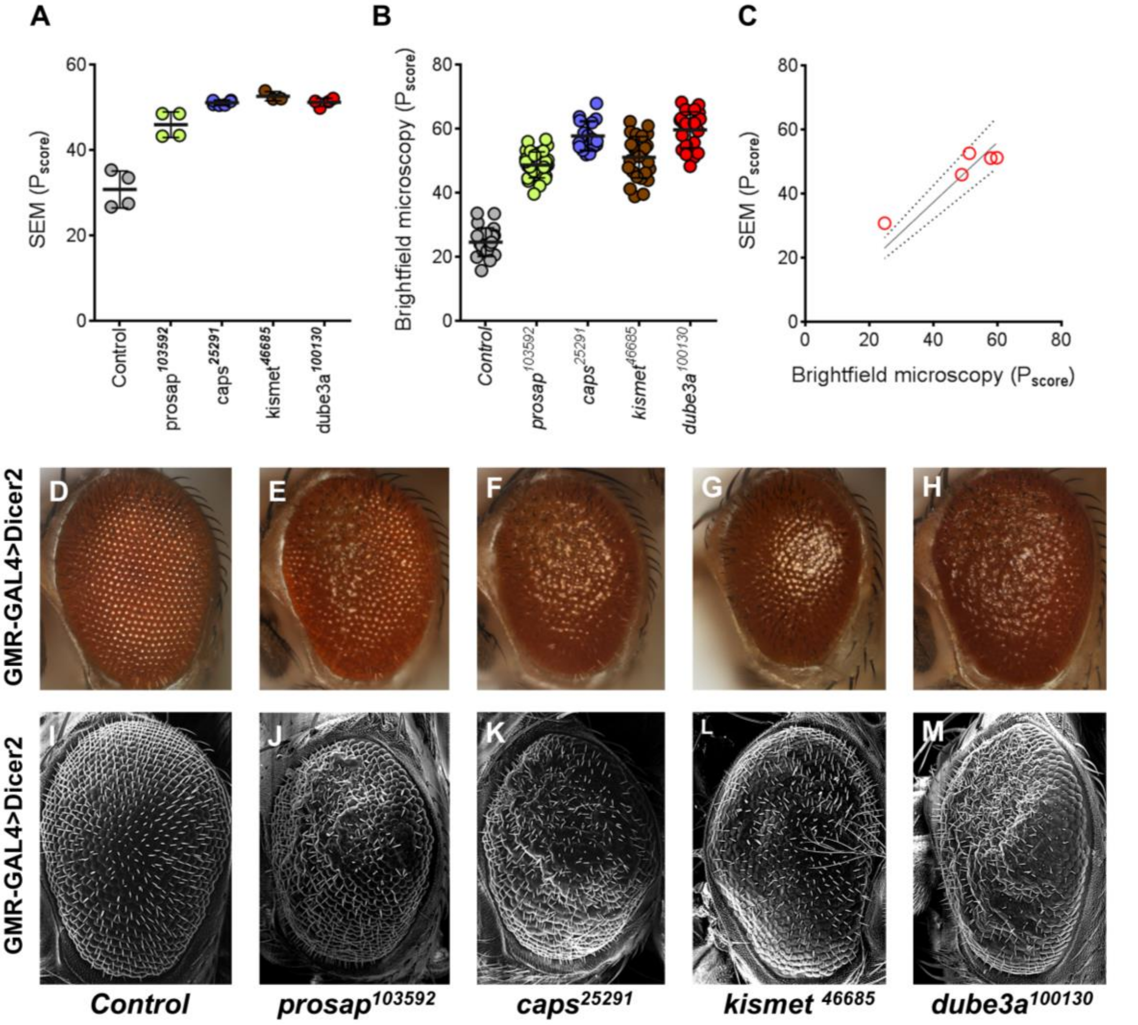

Figure S9. Performance of Flynotyper for SEM and bright field microscope images. Phenotypic scores of GMR-GAL4; Dicer2/+ control flies and fly eyes with GMR-GAL4 driven RNAi knockdown of *prosap*, *caps*, *kismet* and *dube3a* using (**A**) SEM images and (**B**) bright field microscope images are shown. (**C**) A positive correlation is observed (Pearson correlation coefficient to test for linearity, r=0.95, p=0.011) between the scores obtained from SEM compared to bright field microscope. Representative (**D-H**) bright field microscope and corresponding (**I-M**) SEM eye images of GMR-GAL4; Dicer2/+ control flies and fly eyes with RNAi knockdown of *prosap*, *caps*, *kismet* and *dube3a* are also shown. The number of images used for each genotype range from n=4 to n=6 for SEM images and n=7 to n=30 for bright field images.
