# Supplementary material for: Quantitative Assessment of Eye Phenotypes for Functional Genetic Studies Using Drosophila melanogaster
Source: G3 (Bethesda). 2016 Mar 18;6(5):1427–37. doi: 10.1534/g3.116.027060 (PMC4856093; doi:10.1534/g3.116.027060)
Supplement: Supplemental Material [file supp_g3.116.027060_FigureS2.pdf]

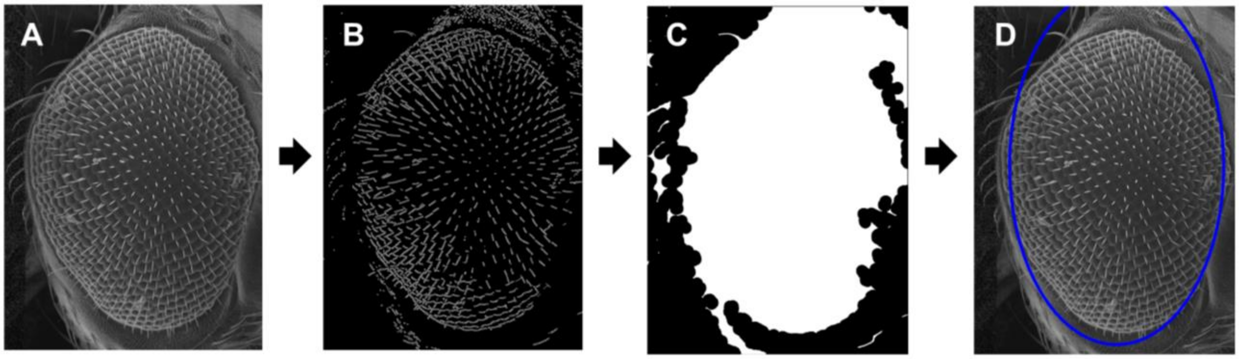

Figure S2. Eye area localization of images obtained from SEM.
Eye localization in SEM image is performed by **(A)** first applying a thresholding operation to the grayscale SEM image, which enables **(B)** better separation of the eye from the background followed by **(C)** edge detection to detect ommatidial cluster region and **(D)** finally closing operation is performed to localize the eye area.
