# Supplementary material for: Quantitative Assessment of Eye Phenotypes for Functional Genetic Studies Using Drosophila melanogaster
Source: G3 (Bethesda). 2016 Mar 18;6(5):1427–37. doi: 10.1534/g3.116.027060 (PMC4856093; doi:10.1534/g3.116.027060)
Supplement: Supplemental Material [file supp_g3.116.027060_FigureS3.pdf]

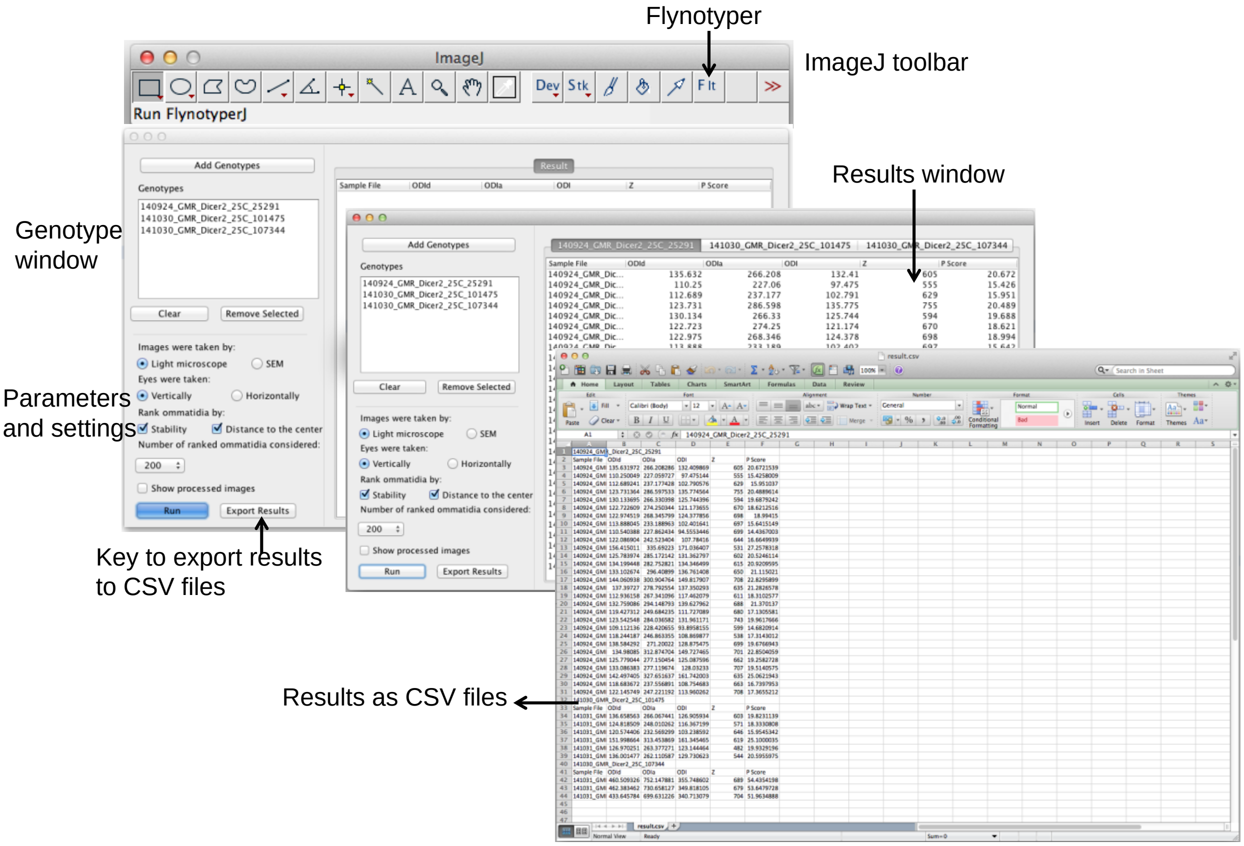

Figure S3. User interface for Flynotyper using the ImageJ plugin.
User interface for imageJ plugin for using Flynotyper shows different components of the software that are user friendly. The parameters and setting can be changed based on the type of microscope used (SEM or bright field), number of ranked ommatidia to be considered for phenotypic score calculation, type of image (vertical or horizontal eye images), and the type of analysis (based on stability or distance to the center). By default, the software will calculate phenotypic scores from the first 200 most-ordered ommatidia. The results window can be further downloaded as a .csv file. On an average, the software can process 1 (1800×2400) image every 3 seconds.
