# Supplementary material for: Quantitative Assessment of Eye Phenotypes for Functional Genetic Studies Using Drosophila melanogaster
Source: G3 (Bethesda). 2016 Mar 18;6(5):1427–37. doi: 10.1534/g3.116.027060 (PMC4856093; doi:10.1534/g3.116.027060)
Supplement: Supplemental Material [file supp_g3.116.027060_FigureS4.pdf]

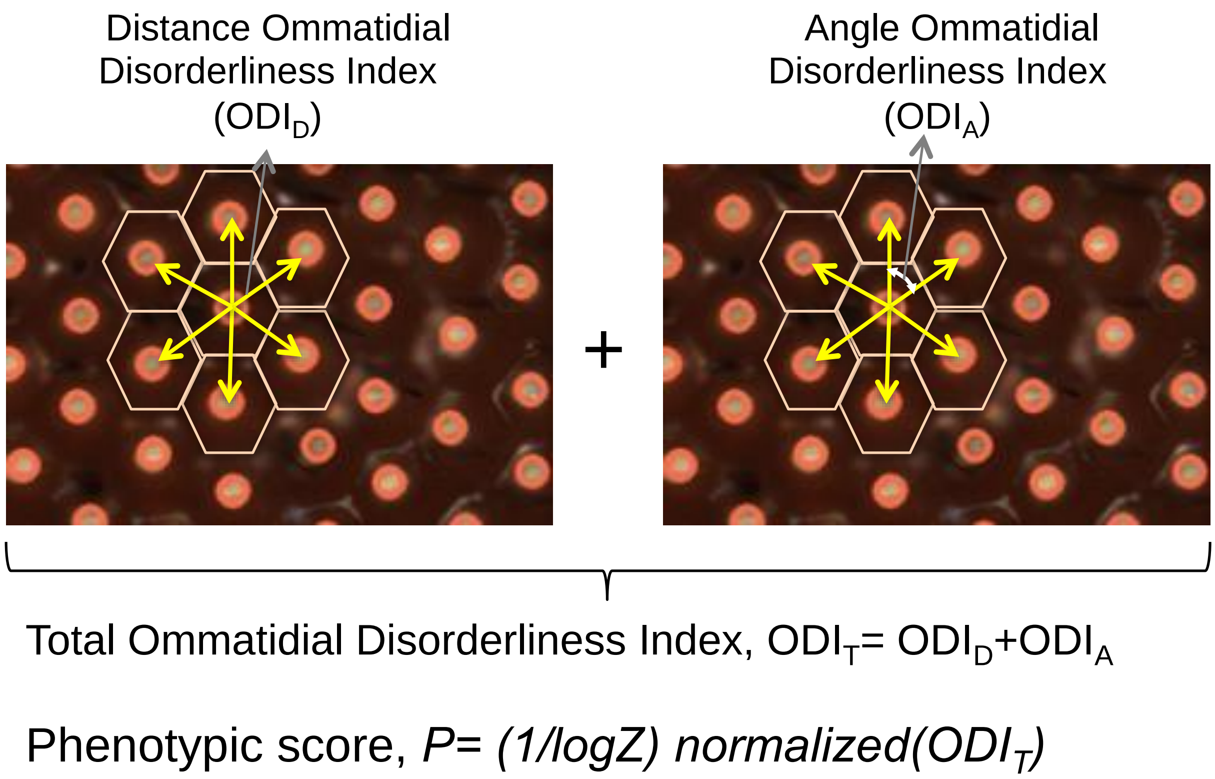

Figure S4. Calculation of phenotypic score.

The figure shows a part of the fly eye with seven ommatidia shown as hexagons in white and the center of ommatidia identified as smaller orange circles within these hexagons. Four metrics are used for the calculation of phenotypic score (ODI_D, ODI_A, ODI_T and fusion index). Six local vectors with direction pointing from each ommatidium to the neighboring ommatidia are shown (yellow arrows). The distance ommatidial disorderliness index, ODI_D, is calculated as the difference between the lengths of each of the five local vectors from the smallest vector. The angle ommatidial disorderliness index, ODI_A, is measured as the difference between the angles formed between pairs of the five local vectors to the smallest angle. The total ommatidial disorderliness index, ODI_T, is the sum of distance and angle ommatidial disorderliness indices using the number of most ordered ommatidia. See Methods and formula (3). Fusion index is the number of ommatidia detected. The sum of the two entropy (disorderliness) measures (ODI_T calculated from ODI_D and ODI_A) and fusion index measures (Z) are used to calculate the phenotypic score using the depicted formula.
