# Supplementary material for: Quantitative Assessment of Eye Phenotypes for Functional Genetic Studies Using Drosophila melanogaster
Source: G3 (Bethesda). 2016 Mar 18;6(5):1427–37. doi: 10.1534/g3.116.027060 (PMC4856093; doi:10.1534/g3.116.027060)
Supplement: Supplemental Material [file supp_g3.116.027060_FigureS5.pdf]

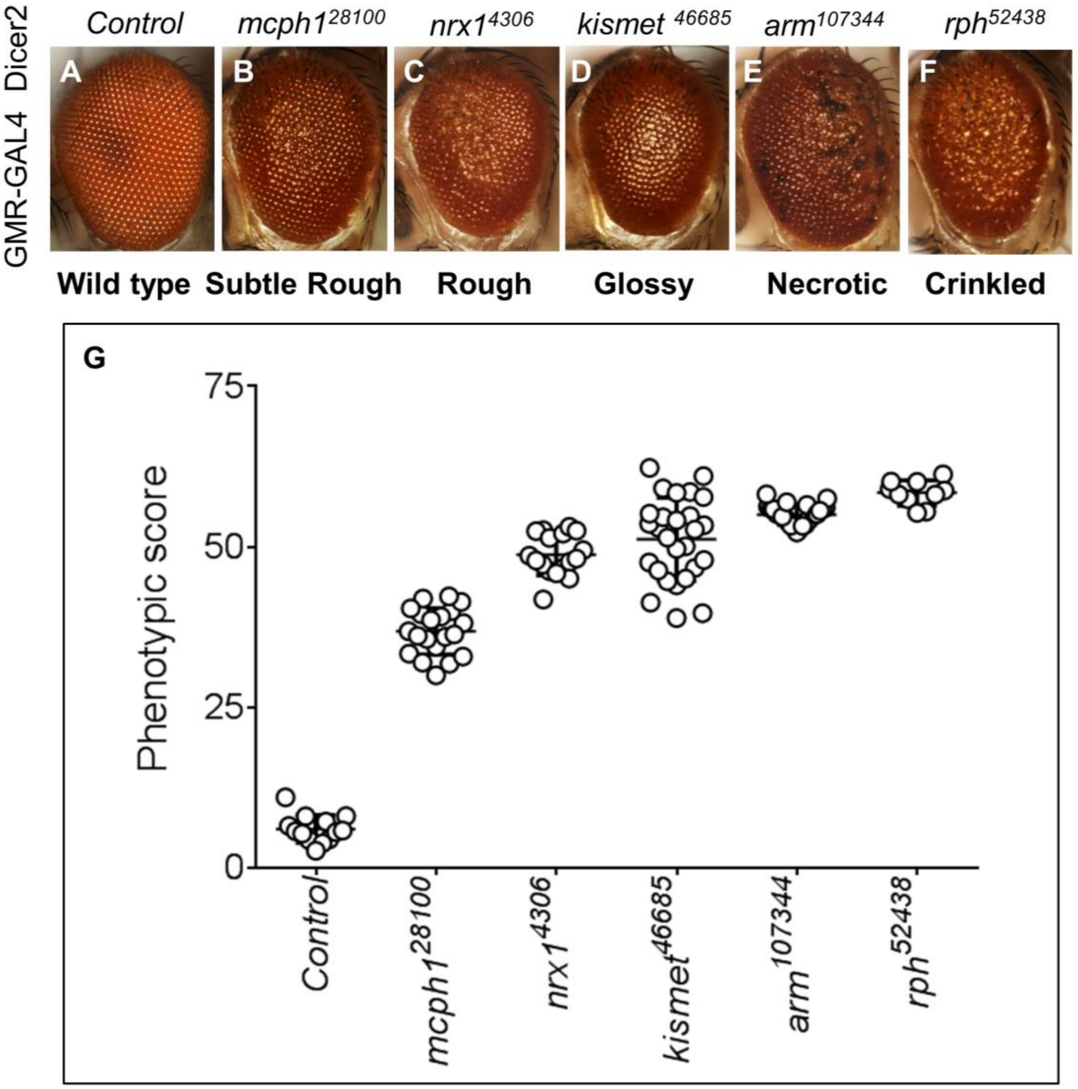

Figure S5. Analysis of different categories of eye phenotypes.
(**A-F**) Representative bright-field microscope eye images displaying eye-specific knockdown of *mcph1*, *nrx-1*, *kismet*, *arm*, and *rph* genes from flies reared at 30°C. Eyes of GMR-GAL4; Dicer2/+ control flies show normal ommatidial organization, while the eyes of flies with GMR-GAL4 driven RNAi knockdown of the 5 genes show disruption in the morphology of the eye that can be classified into different categories. (**G**) Graph representing the phenotypic scores of control flies compared to 5 different categories of eye morphology. The number of images analyzed for each of these genotypes is as follows: control (n=22), *mcph1* (n=20), *nrx-1* (n=17), *kismet* (n=27), *arm* (n=25), and *rph* (n=10).
