# Supplementary material for: Quantitative Assessment of Eye Phenotypes for Functional Genetic Studies Using Drosophila melanogaster
Source: G3 (Bethesda). 2016 Mar 18;6(5):1427–37. doi: 10.1534/g3.116.027060 (PMC4856093; doi:10.1534/g3.116.027060)
Supplement: Supplemental Material [file supp_g3.116.027060_FigureS6.pdf]

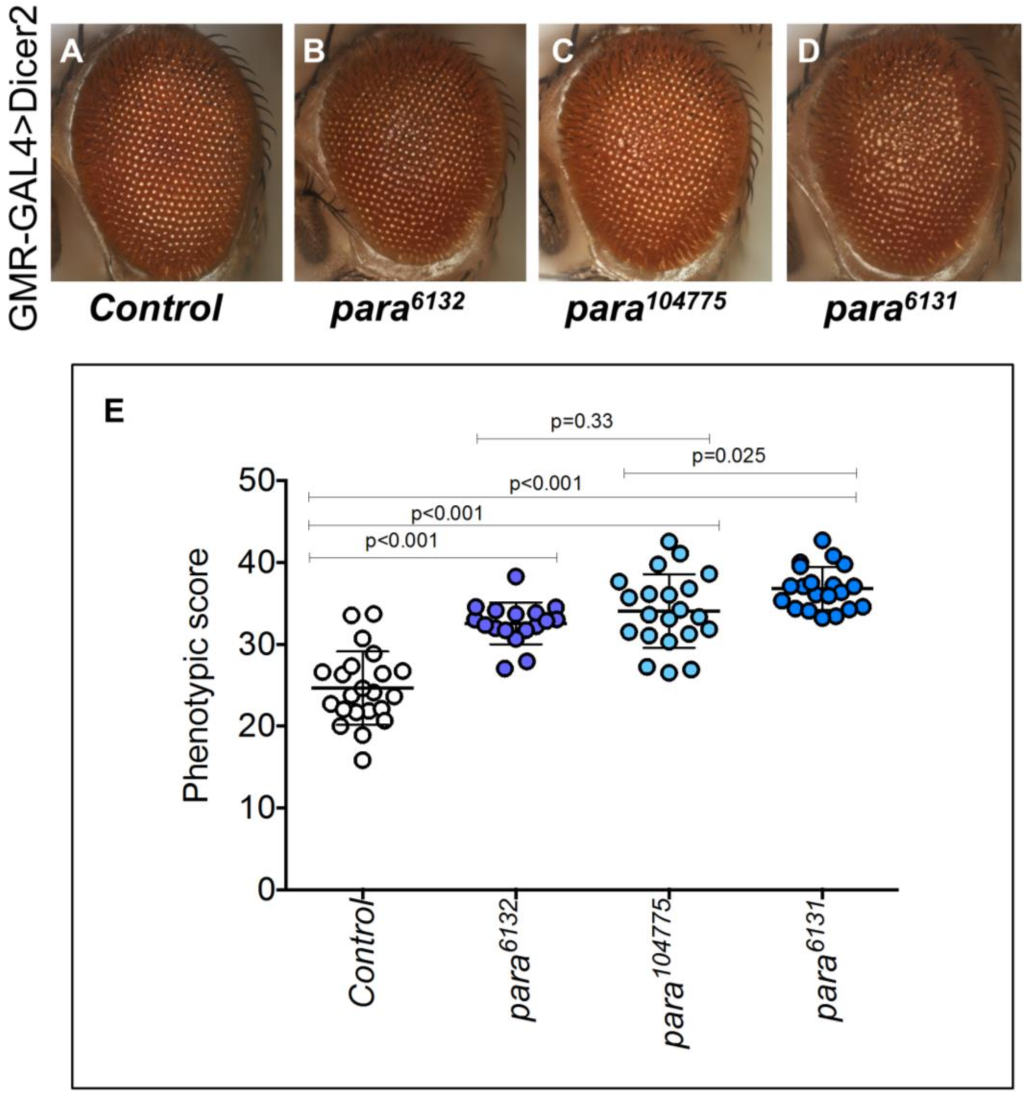

Figure S6. Phenotypic analysis of fly lines with RNAi mediated knockdown of para (*SCN1A* in humans).

(**A-D**) Representative bright-field microscope images of fly eyes displaying eye specific knockdown (using GMR-GAL4) of *para* in three different RNAi lines, $para^{6131}$, $para^{6132}$ and $para^{104775}$ are shown. (**E**) Graphs representing the phenotypic scores of control lines (wVDRC) and three fly lines with *para* knockdown are also shown. Phenotypic scores of these three RNAi lines enabled us to accurately distinguish between the subtle phenotypic changes. Asterisks (*) represent significant difference by student *t* test (Mann Whitney test, two-tailed $p<0.001$). The number of images analyzed is as follows: controls (n=22), $para^{6131}$ (n=20), $para^{6132}$ (n=17) and $para^{104775}$ (n=21).
