# Supplementary material for: Quantitative Assessment of Eye Phenotypes for Functional Genetic Studies Using Drosophila melanogaster
Source: G3 (Bethesda). 2016 Mar 18;6(5):1427–37. doi: 10.1534/g3.116.027060 (PMC4856093; doi:10.1534/g3.116.027060)
Supplement: Supplemental Material [file supp_g3.116.027060_FigureS7.pdf]

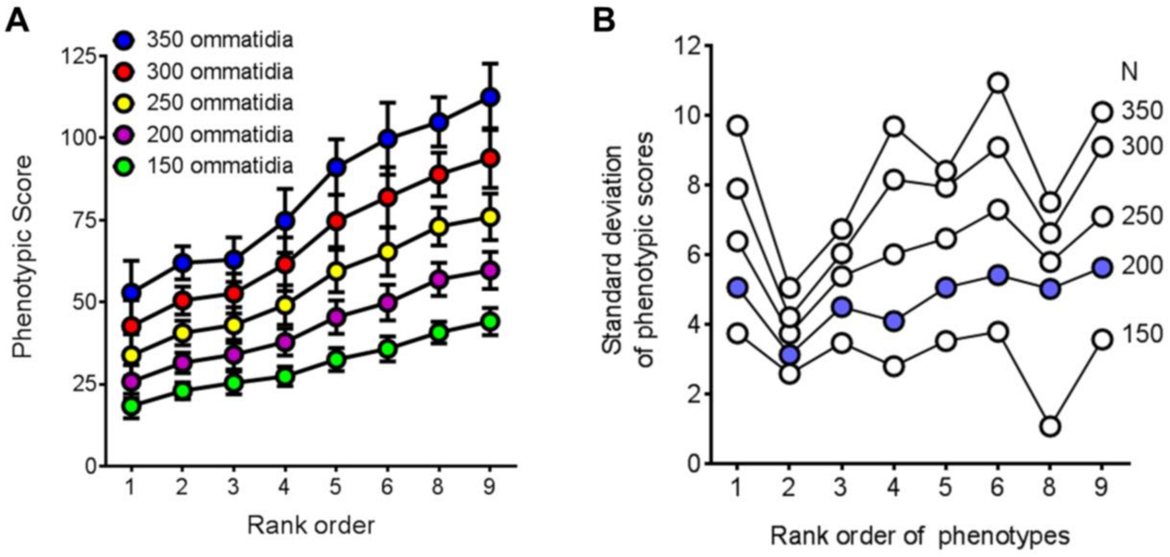

Figure S7. Performance of Flynotyper at different ommatidial counts (N).
(**A**) A graph representing phenotypic scores of genotypes across manually determined ranks (for flies reared at 30°C) for different numbers of ommatidia is shown. The phenotypic scores across the manual ranks ranging from 1 to 9 follow the same trend at different ommatidial counts, reflecting the robustness of our method. (**B**) The variability (denoted by standard deviation) of phenotypic scores at different counts of ommatidia (at N=100, 200, 250, 300, and 350) is shown. The variation is the least for N=200 for genotypes within the same rank or across different rank orders and was adopted as the default parameter in the Flynotyper software for calculation of phenotypic scores. We also note that the user can chose anywhere between 50 and 350, but making sure that this number (N) is smaller than the total number of ommatidia, for calculation of phenotypic scores. For smaller sized eyes, users can choose N as few as 50. N is standard and should be selected only once for all eyes tested in the same experiment. Comparison (or combining) of phenotypic scores across experiments should be performed only if the same number of ommatidia (N) was selected for each of the experiments.
