# Supplementary material for: Quantitative Assessment of Eye Phenotypes for Functional Genetic Studies Using Drosophila melanogaster
Source: G3 (Bethesda). 2016 Mar 18;6(5):1427–37. doi: 10.1534/g3.116.027060 (PMC4856093; doi:10.1534/g3.116.027060)
Supplement: Supplemental Material [file supp_g3.116.027060_FigureS8.pdf]

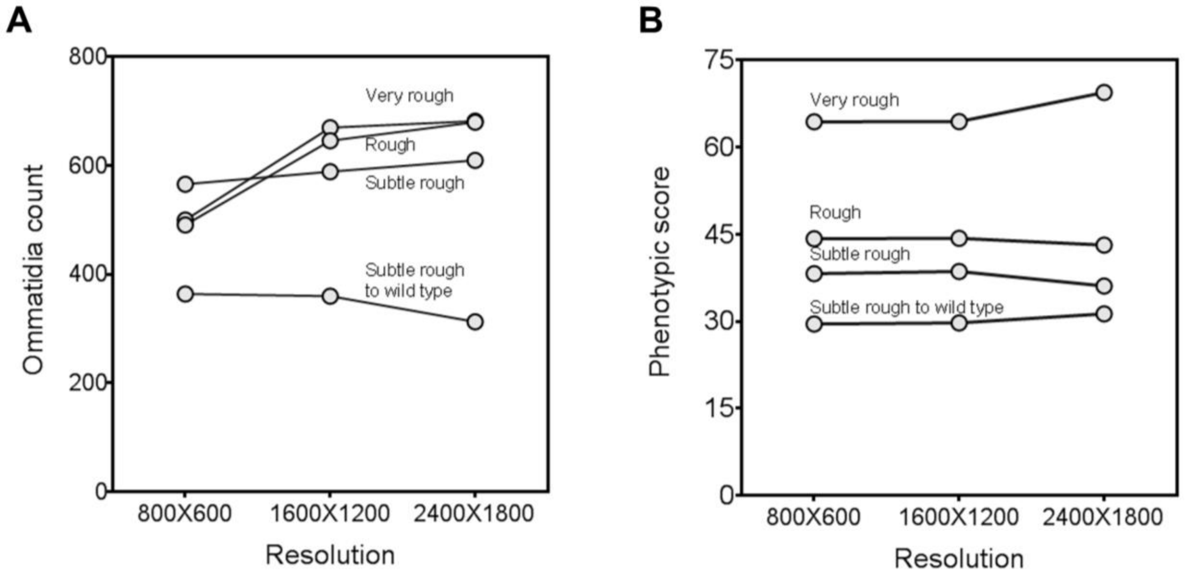

Figure S8. Performance of Flynotyper at image resolutions is shown.

(**A**) Number of ommatidia detected at different image resolutions is shown. (**B**) Phenotypic score calculation at different image resolutions is shown. Note that although the number of ommatidia detected increases with higher resolution images (1200×1600 and 1600×2400), the phenotypic scores are unaltered.
