# Supplementary material for: Quantitative Assessment of Eye Phenotypes for Functional Genetic Studies Using Drosophila melanogaster
Source: G3 (Bethesda). 2016 Mar 18;6(5):1427–37. doi: 10.1534/g3.116.027060 (PMC4856093; doi:10.1534/g3.116.027060)
Supplement: Supplemental Material [file supp_g3.116.027060_FigureS10.pdf]

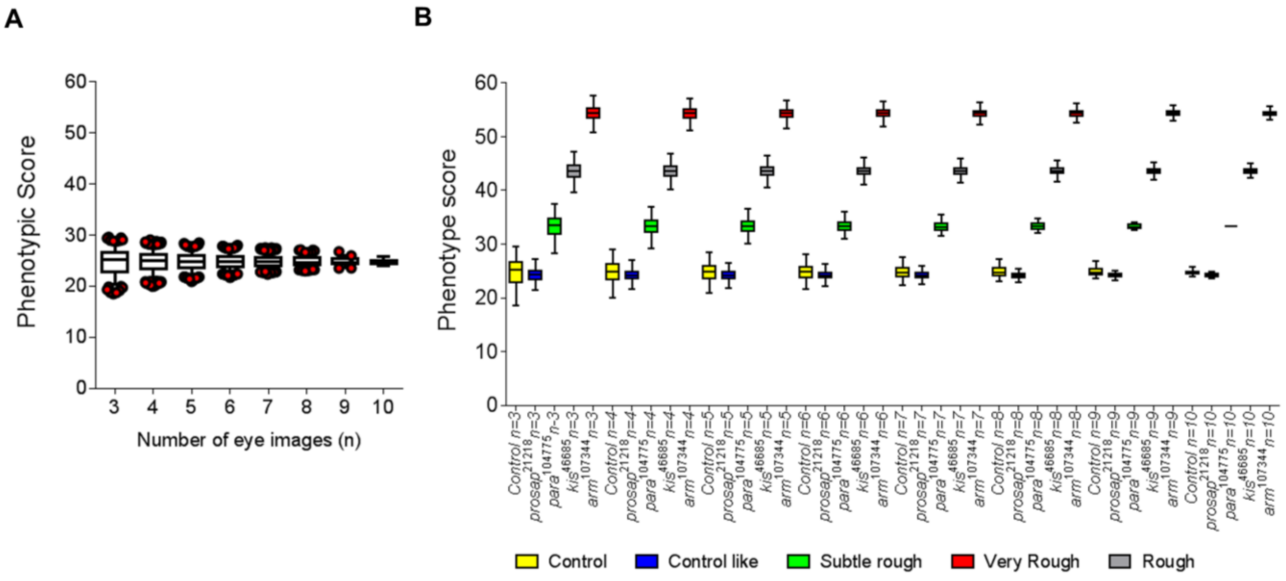

Figure S10. A test for sensitivity of Flynotyper and its ability to distinguish between different classes of phenotypes.

(**A**) The plot shows the phenotypic scores when combinations ($^{10}C_3$ number of combinations) of eye images at n=3 to n=10 are tested. Each box plot shows the distribution of mean phenotypic score (mean and SD) for each combination of eye images. Note that the distribution is tighter for larger numbers of eye images. (**B**) Distribution of mean phenotypic scores for each combination of n=3 to n=10 images were tested. Each of the fly eye phenotypic categories was tested against each other using a Mann Whitney test. While no difference in the distribution of mean phenotypic scores (for n=3 to n=10) was observed between control and control like eye images, significant differences were observed for each of the other categories compared to each other and with the control eye images (two-tailed $p<0.001$, Mann Whitney test).
