# Supplementary material for: Quantitative Assessment of Eye Phenotypes for Functional Genetic Studies Using Drosophila melanogaster
Source: G3 (Bethesda). 2016 Mar 18;6(5):1427–37. doi: 10.1534/g3.116.027060 (PMC4856093; doi:10.1534/g3.116.027060)
Supplement: Supplemental Material [file supp_g3.116.027060_FigureS11.pdf]

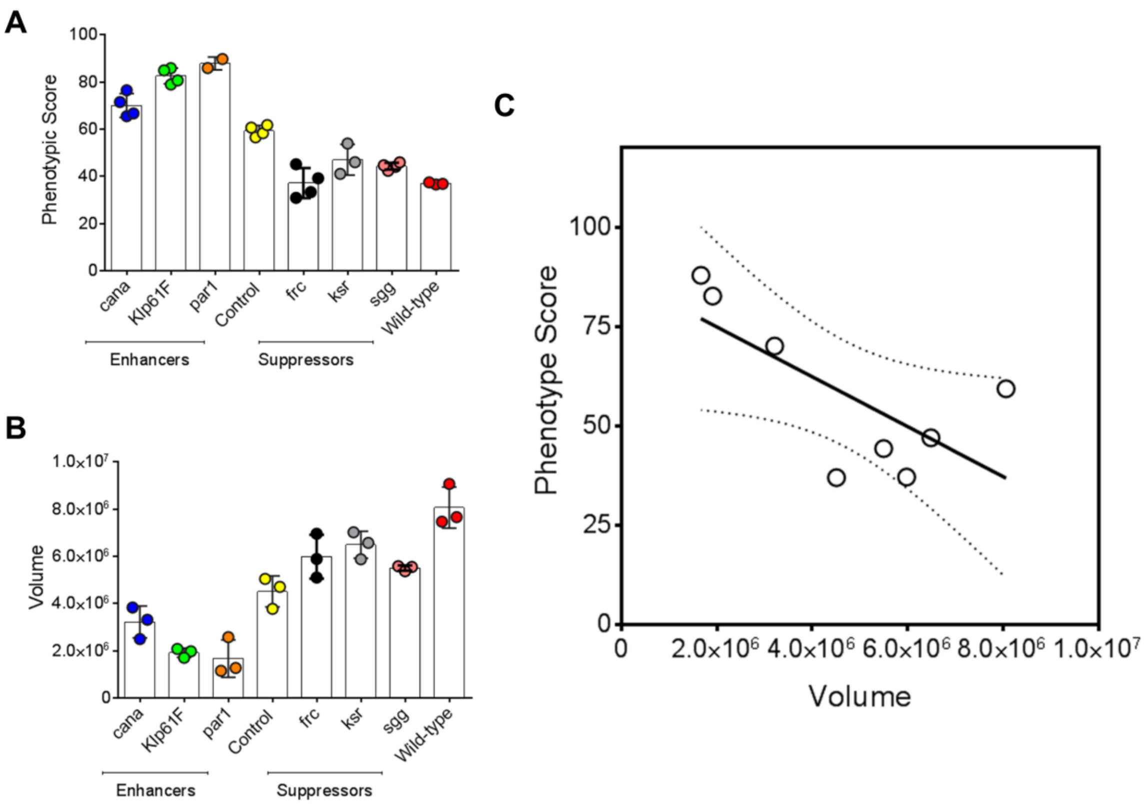

Figure S11. Validation of Flynotyper for images obtained from independent studies (Example 1).

Example of validation using data from Ambegaokar and Jackson (Ambegaokar and Jackson 2011). **(A)** A graph representing the phenotypic scores of wild-type, control, and the three enhancers and suppressors of *w*^{1118}/+;*gl-tau*/+ is shown. The control listed is *w*^{1118}/+;*gl-tau*/+. All other panels, except wld-type, contain one copy of *gl-tau* transgene *in trans* to one disrupted copy of the gene listed in the panel. **(B)** A graph representing the eye volumes of wild-type, control, and the three enhancers and suppressors of *w*^{1118}/+;*gl-tau*/+ is shown. **(C)** Correlation plot of phenotypic scores and eye volumes shown significantly negative correlation between the volume of the eyes and the phenotypic scores (Pearson r=-0.71, two-tailed p=0.049). These observations suggest that the phenotypic scores for the modifiers are concordant with the original assessment. The number of images used for these analyses were n=2 for par1, n=3 each for wild type and ksr, n=4 each for control, cana, Klp61F, sgg, and frc.
