# Supplementary material for: Quantitative Assessment of Eye Phenotypes for Functional Genetic Studies Using Drosophila melanogaster
Source: G3 (Bethesda). 2016 Mar 18;6(5):1427–37. doi: 10.1534/g3.116.027060 (PMC4856093; doi:10.1534/g3.116.027060)
Supplement: Supplemental Material [file supp_g3.116.027060_FigureS12.pdf]

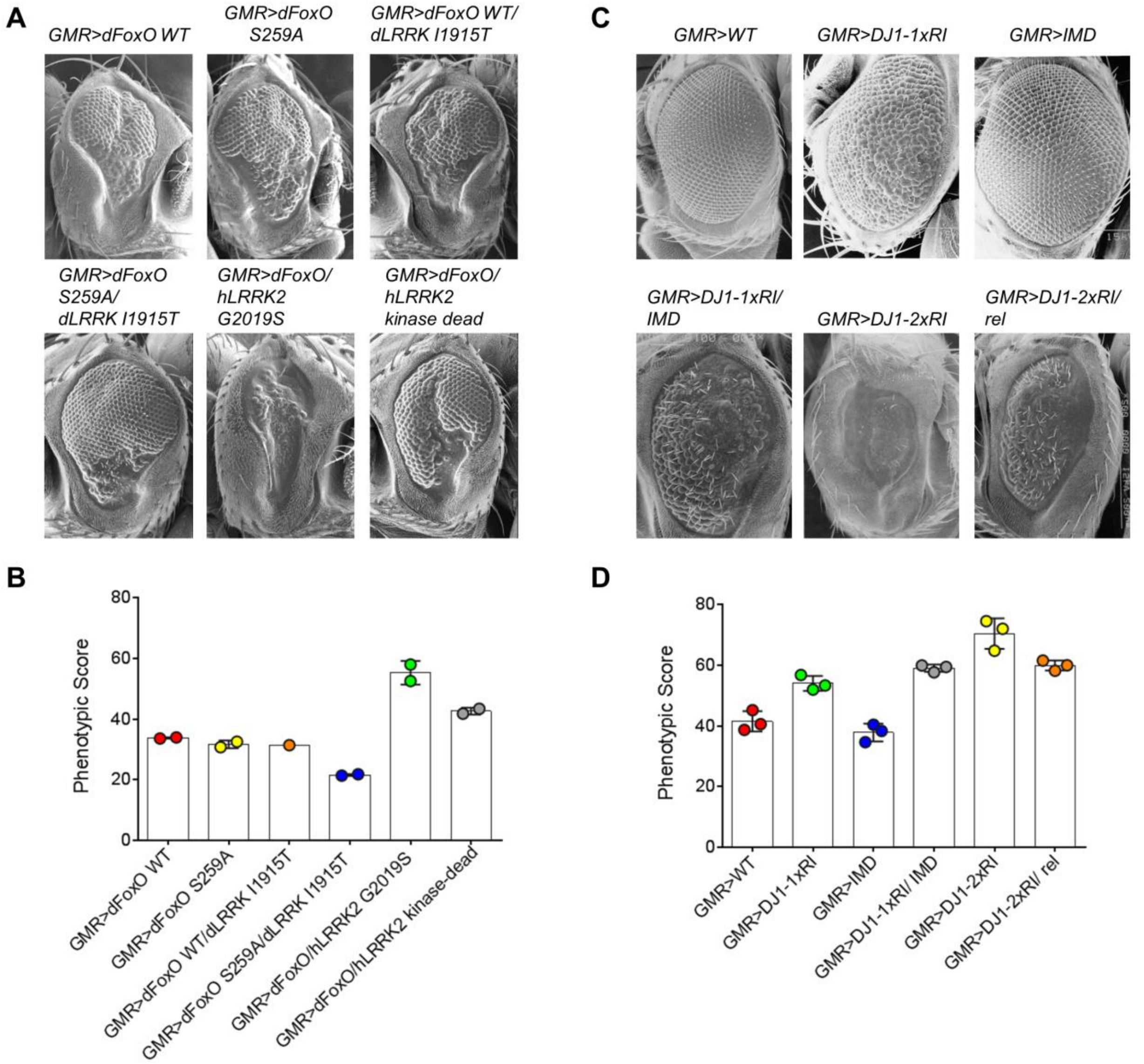

Figure S12. Validation of Flynotyper for images obtained from independent studies (Example 2).

(A) High resolution SEM images of interactors of *dFoxO*. While, the eye degeneration phenotype of *dFoxO S259A* mutation is partially rescued by *dLRRK I1915T*, *hLRRK2 G2019S and hLRRK2 kinase dead* mutations enhance the eye phenotype caused by expression of *dFoxO*. **(B)** A graph representing the phenotypic scores of interactors of *dFoxO* using the GMR-GAL4 driver is shown. The phenotypic scores are concordant with the visual assessment of the eye phenotypes, showing that *LRRK* and *dFoxO* are interactors. The number of images processed were n=1 for two genotypes and n=2 for the remaining six genotypes. **(C)** SEM images of RNAi knockdown of *DJ-1* and its modifiers. While IMD over expression by itself has no effect on fly eye morphology, it enhanced the rough-eye phenotype caused by moderate *DJ-1* RNAi (*DJ1-*

1xRI: one copy of the *DJ-1* RNAi transgene driven by GMR-Gal4). On the other hand, loss of one copy of *relish* (*rel*/+), a key component in the IMD pathway, partially rescued the strong eye phenotype caused by over expression of 2 copies of the *DJ-1* RNAi transgene (*DJ1-2xRI*). (**D**) A graph representing the phenotypic scores of modifiers of *DJ-1* RNAi using the GMR-GAL4 driver is shown. The phenotypic scores are concordant with the visual assessment of the eye phenotypes and suggest that *IMD* and *rel* interact with *DJ-1*. The number of images processed were n=3 for all the samples.
