# Supplementary material for: Quantitative Assessment of Eye Phenotypes for Functional Genetic Studies Using Drosophila melanogaster
Source: G3 (Bethesda). 2016 Mar 18;6(5):1427–37. doi: 10.1534/g3.116.027060 (PMC4856093; doi:10.1534/g3.116.027060)
Supplement: Supplemental Material [file supp_g3.116.027060_FigureS13.pdf]

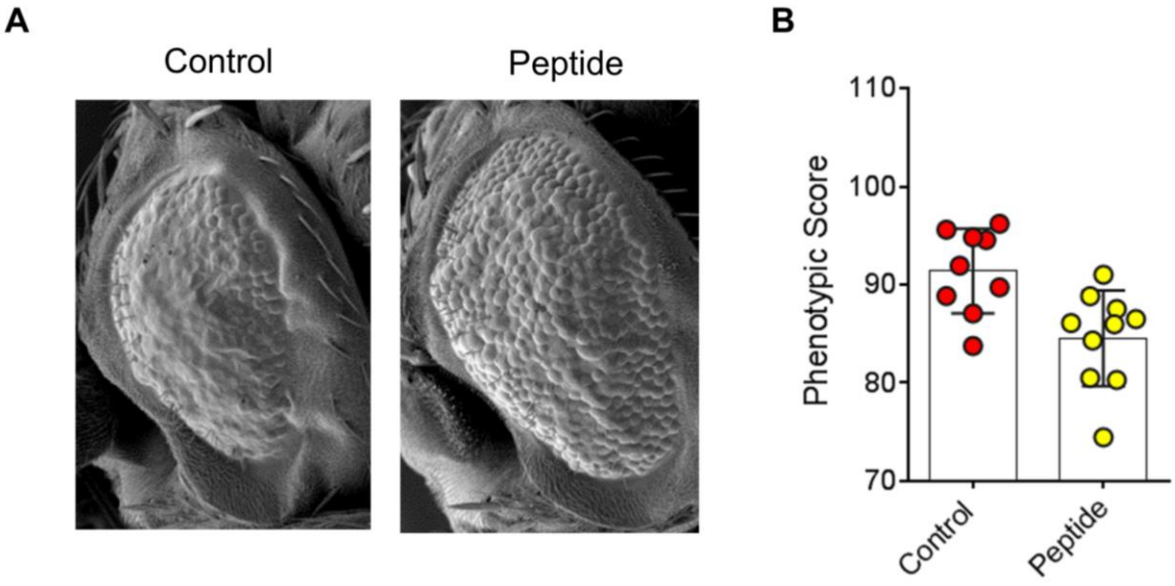

Figure S13. Suppression of UAS-i(CTG)480 toxicity by transgenic expression of ABP1 peptide.

(A) Representative SEM eye images of control (GMR-Gal4>*UAS-i(CTG)$_{480}$UAS-GFP*) and peptide (GMR-Gal4>*UAS-i(CTG)$_{480}$UAS-ABP1*). Expression of *UAS-i(CTG)$_{480}$* caused roughness and reduced eye size. This was significantly suppressed with *UAS-ABP1* peptide expression. **(B)** A graph representing the phenotypic scores of control and peptide treatment. The phenotypic scores are concordant with the visual assessment and show that the ABP1 peptide treatment suppresses the eye phenotype caused by expression of *UAS-i(CTG)$_{480}$.* The number of images processed were n=9 for controls and n=10 for peptide treated eyes.
