# Supplementary material for: Quantitative Assessment of Eye Phenotypes for Functional Genetic Studies Using Drosophila melanogaster
Source: G3 (Bethesda). 2016 Mar 18;6(5):1427–37. doi: 10.1534/g3.116.027060 (PMC4856093; doi:10.1534/g3.116.027060)
Supplement: Supplemental Material [file supp_g3.116.027060_FigureS14.pdf]

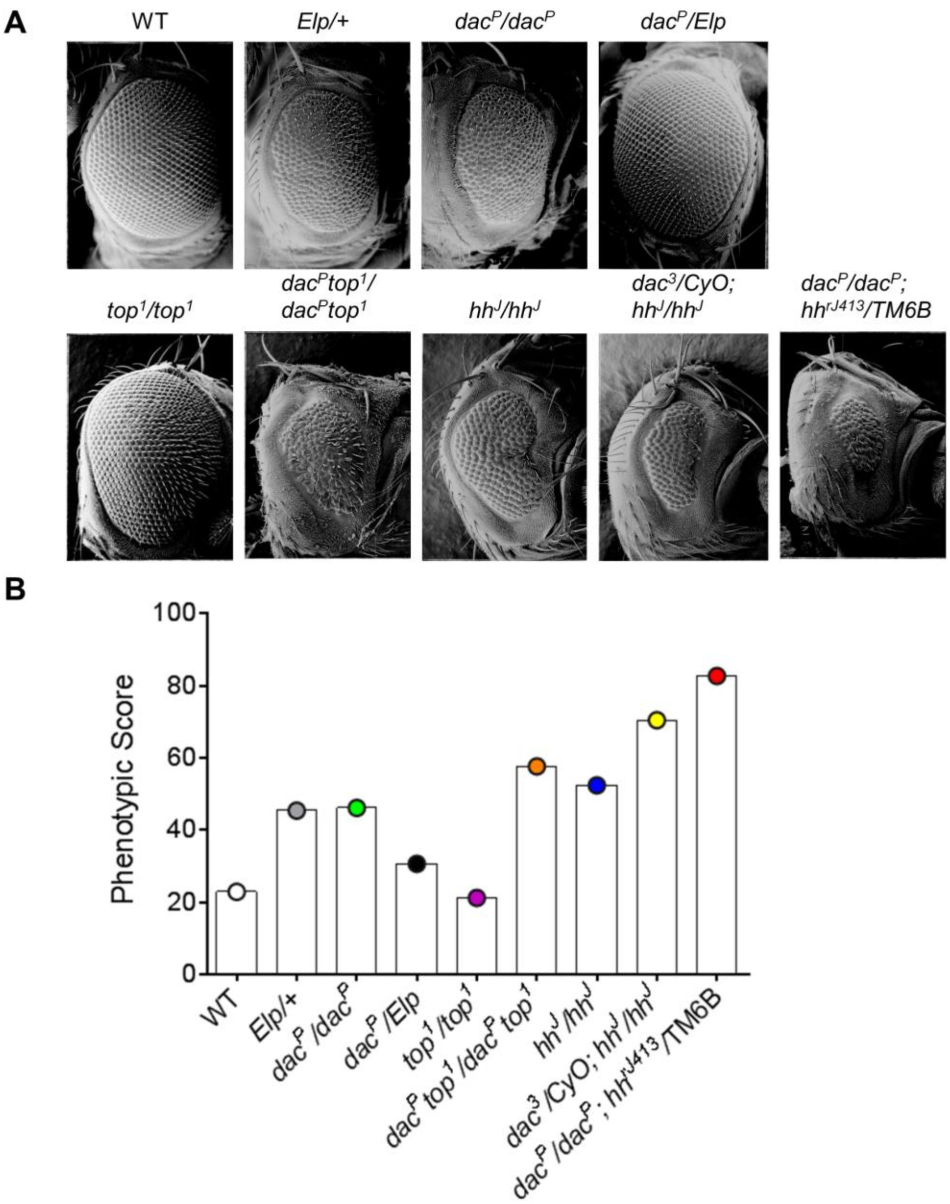


**Figure S14. A genetic screen for interactors of Egfr using P-element insertions.** (**A**) High resolution SEM images of interactors of *Egfr* and *dac*. While *Elp* (a dominant allele of *Egfr*) heterozygote that has rough eyes, is suppressed by a single copy of $dac^P$, $top^1$, a weak allele of *Egfr*, enhances the $dac^P$ rough eye phenotype. Similarly, the $dac^P$ rough eye phenotype is enhanced *by* $hh^{rJ413}$ allele of hedgehog gene. Additionally, $hh^J$ allele enhances $dac^3$ rough eye phenotype. These results indicate interaction between *Egfr* and *dac* and also *dac* and *hh*. (**B**) A graph representing the phenotypic scores of the interactors of *Egfr* and *dac*. The phenotypic scores also confirm the interaction between *Egfr* and *dac* and also *dac* and *hh.*
