# Supplementary material for: Quantitative Assessment of Eye Phenotypes for Functional Genetic Studies Using Drosophila melanogaster
Source: G3 (Bethesda). 2016 Mar 18;6(5):1427–37. doi: 10.1534/g3.116.027060 (PMC4856093; doi:10.1534/g3.116.027060)
Supplement: Supplemental Material [file supp_g3.116.027060_FigureS15.pdf]

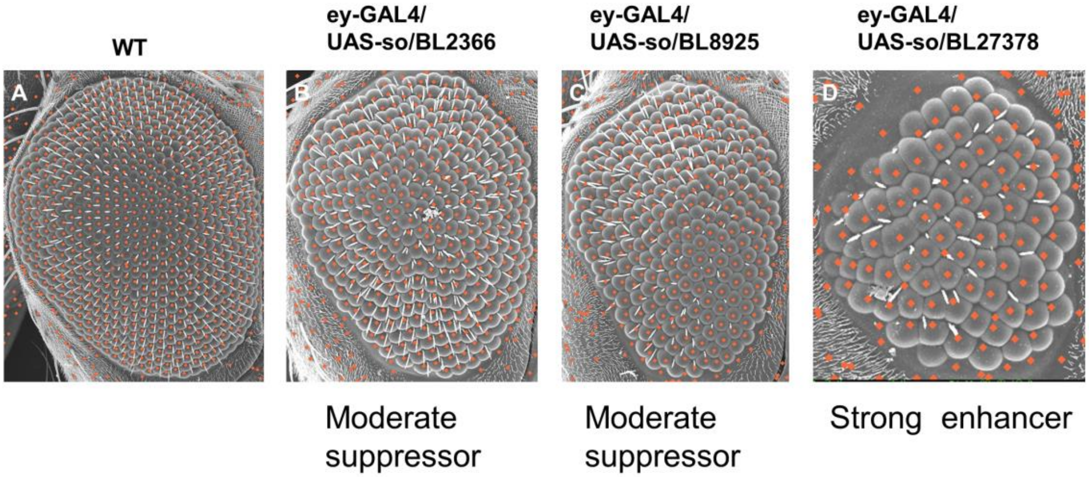

Figure S15. Flynotyper analysis of SEM images to identify modifiers of *sine oculis*. Representative SEM eye images of wild type **(A)** and genetic modifiers of *sine oculis* (*so*) (**B**-**D**), displaying their ommatidial centers (red dots) as detected by Flynotyper are shown.
