# Supplementary material for: Quantitative Assessment of Eye Phenotypes for Functional Genetic Studies Using Drosophila melanogaster
Source: G3 (Bethesda). 2016 Mar 18;6(5):1427–37. doi: 10.1534/g3.116.027060 (PMC4856093; doi:10.1534/g3.116.027060)
Supplement: Supplemental Material [file supp_g3.116.027060_FigureS16.pdf]

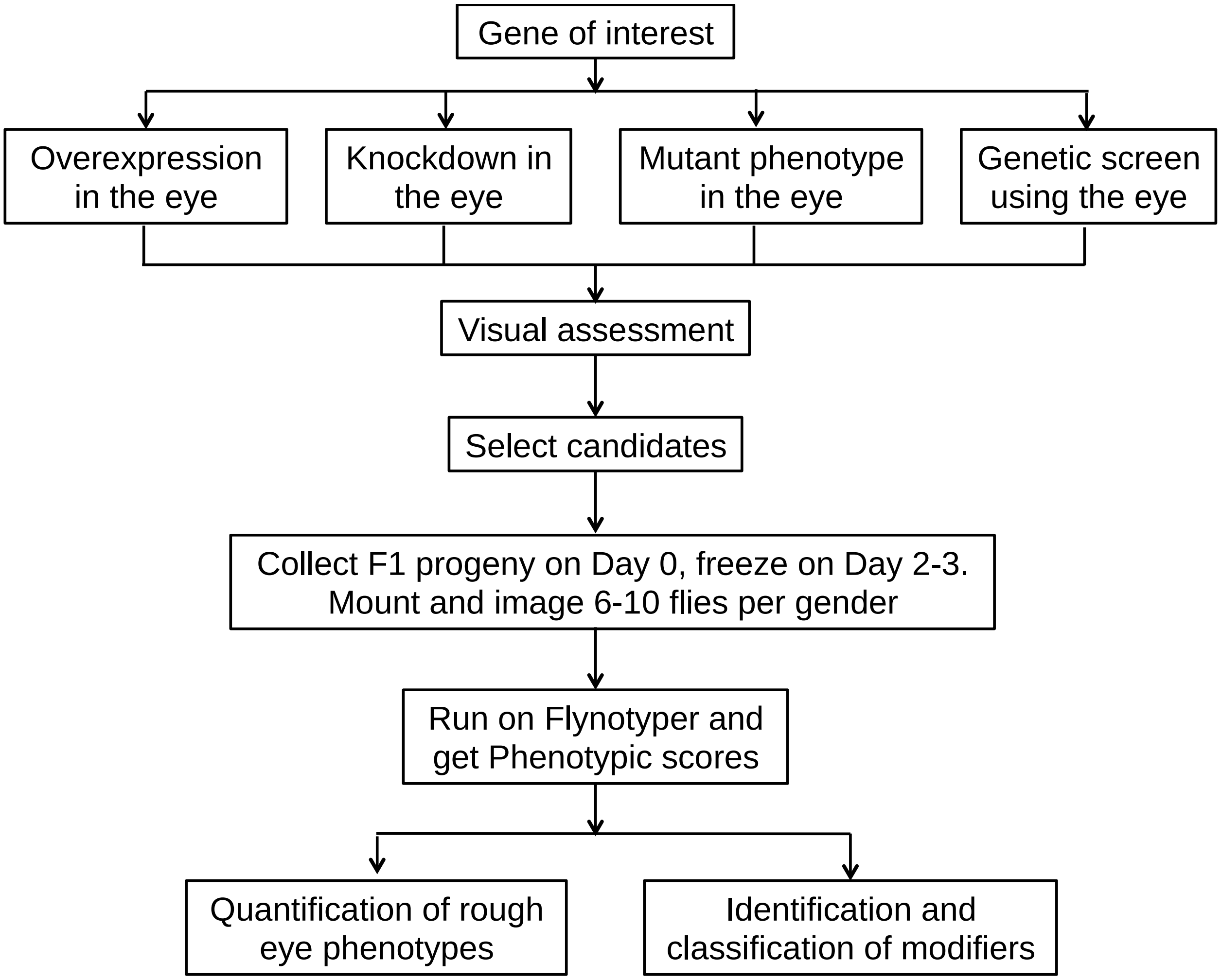

Figure S16. Flowchart depicting the utility of Flynotyper.
A schematic for the proposed use of Flynotyper is shown. Flynotyper can be used for understanding the effects of genetic mutations, gene-dosage alteration, and modifier genes. For genome-wide modifier screens, usually visual inspection under the microscopy is used to identify a subset of candidates that is further classified as enhancers and suppressors using Flynotyper. The method is automated in that the images do not need to be delineated for the region of interest. The Flynotyper software will automatically detect the fly eye and process each image for phenotype score.
